# Glycome Profiling of Cancer Cell Lines Cultivated in Physiological and Commercial Media

**DOI:** 10.3390/biom12060743

**Published:** 2022-05-24

**Authors:** Junyao Wang, Wenjing Peng, Aiying Yu, Mohamed Fokar, Yehia Mechref

**Affiliations:** 1 Department of Chemistry and Biochemistry, Texas Tech University, Lubbock, TX 79409, USA; junyao.wang@ttu.edu (J.W.); wenjing.peng@ttu.edu (W.P.); aiying.yu@ttu.edu (A.Y.); 2 Center of Biotechnology and Genomics, Texas Tech University, Lubbock, TX 79409, USA; m.fokar@ttu.edu

**Keywords:** glycomics, culturing media, cancer cell line, differential expression analysis, LC-MS/MS

## Abstract

A complex physiological culture medium (Plasmax) was introduced recently, composed of nutrients and metabolites at concentrations normally found in human plasma to mimic the in vivo environment for cell line cultivation. As glycosylation has been proved to be involved in cancer development, it is necessary to investigate the glycan expression changes in media with different nutrients. In this study, a breast cancer cell line, MDA-MB-231BR, and a brain cancer cell line, CRL-1620, were cultivated in Plasmax and commercial media to reveal cell line glycosylation discrepancies prompted by nutritional environments. Glycomics analyses of cell lines were performed using LC-MS/MS. The expressions of multiple fucosylated *N*-glycans, such as HexNAc_4_Hex_3_DeoxyHex_1_ and HexNAc_5_Hex_3_DeoxyHex_1_, derived from both cell lines exhibited a significant increase in Plasmax. Among the *O*-glycans, significant differences were also observed. Both cell lines cultivated in EMEM had the lowest amounts of *O*-glycans expressed. The original work described the development of Plasmax, which improves colony formation, and resulted in transcriptomic and metabolomic alterations of cancer cell lines, while our results indicate that Plasmax can significantly impact protein glycosylation. This study also provides information to guide the selection of media for in vitro cancer cell glycomics studies.

## 1. Introduction

The nutrient compositions of culture media can profoundly affect the phenotypic behavior of cells, including their response to stress and stimuli, the epigenotype. However, most of the commercially available and widely used cell culture media may not be able to mimic the in vivo environment for cell lines. The compositions of the commercial media were not designed to reproduce a physiological cellular environment, such as human plasma, but rather to provide continuous cultivation of cells using a minimal amount of nutrients. A good example is Eagle’s Minimal Essential Medium (EMEM), a synthetic cell culture medium developed by Harry Eagle [1]. In addition to EMEM, some media were modified by increasing the concentration of selected nutrients to avoid nutrient exhaustion for a longer duration of cultivation without attendance [2]. For example, Dulbecco’s Modified Eagle’s Medium (DMEM) was introduced with higher concentrations of amino acids and vitamins [3]. EMEM and DMEM are both widely used in cell culturing [4,5,6]. However, the high concentration of certain nutrients can result in metabolic alterations between in vitro and in vivo cancer cells [7]. Recently, efforts have been directed to optimize the compositions and concentrations of culture media for cancer cells [8,9,10,11]. A complex culture medium, Plasmax, was introduced by Voorde et al. [12], which is composed of more than 60 nutrients and metabolites, of which 35% are not included in DMEM or EMEM. Thus, Plasmax provides more nutritional options. Moreover, a variety of elements such as selenium and pyruvate, which endogenously exist in human plasma and are important for cancer cell progression, are also included in Plasmax. In order to minimize the gap between in vitro and in vivo environments and reproduce the in vivo environment for cancer cell cultivation, the concentration of each nutrient was maintained at the physiological level of human blood. When breast cancer cell lines were cultured in Plasmax and DMEM, the colony-forming capacity of the cancer cells was enhanced in Plasmax relative to DMEM due to the micronutrient selenium, which can prevent ferroptosis [12]. In addition, the transcriptional and metabolic phenotypes of cancer cells cultivated by Plasmax were found to be independent of the proliferation rate. This suggests that the high concentration of some nutrients in commercial media is not required for cell growth and could misdirect the metabolism. Plasmax provides a better culturing environment for cancer cell lines [12]. However, the influence of such a plasma-like medium on glycosylation has not yet been assessed.

Glycosylation is one of the most common post-translational modifications (PTMs), which plays an important role in cellular activity regulation [13,14]. For human proteins, more than 50% are glycosylated [15]. Glycans can serve a variety of functional and structural roles in the membrane and secreted proteins, including protein stabilization and solubilization, cell–cell interactions, cell adhesion, pathogen interactions, and cellular immune recognition [16,17,18]. Post-translational glycosylation is also one of the important epigenetic changes that occur during neoplastic transformation [19]. For example, expression of STn (a truncated *O*-glycan containing a sialic acid α-2,6 linked to GalNAc α-*O*-Ser/Thr) antigen can inhibit cell adhesion and thus increase cell movement and spread [20,21]. Higher expression of GnT-V-dependent *N*-glycan leads to an enhancement of the invasion of glioma, colon cancer, and gastric cancer cell lines [22,23]. Recent studies also unveiled the connections between glycan expressions and chronic kidney diseases (CKD) [24,25]. Therefore, it is necessary to assess the glycan expression alterations in Plasmax to better understand the influence of nutrients in different media on cell glycosylation.

In this study, we reformulated the complex medium, Plasmax, and compared the glycosylation of proteins in this media to two common commercial media, DMEM and EMEM. One breast cancer cell line (MDA-MB-231BR) and one brain cancer cell line (CRL-1620) were cultivated in Plasmax, DMEM, and EMEM. The *N*- and *O*-linked glycans of these two cell lines cultivated in the three different culturing media were extracted, permethylated, and analyzed using nano-LC-MS/MS. The relative abundance of glycans was calculated and compared to assess how nutrients in different media impact glycan expressions in different cancer cell lines.

## 2. Materials and Methods

### 2.1. Materials and Reagents

Breast cancer cell line MDA-MB-231BR was generously provided by Dr. Paul Lockman (West Virginia University, School of Pharmacy, Morgantown, WV, USA). Brain cancer cell line CRL-1620, DMEM, EMEM, phosphate-buffered saline (PBS), and fetal bovine serum (FBS) were purchased from American Type Culture Collection (ATCC, Manassas, VA, USA). Trypsin-ethylenediaminetetraacetic acid (EDTA) 1× (25% Trypsin/2.21 mM EDTA), formic acid (FA), HPLC-grade water, methanol, and acetonitrile were purchased from Fisher Scientific (Fair Lawn, NJ, USA). Dimethyl sulfoxide (DMSO), sodium hydroxide beads, iodomethane, pronase from Streptomyces griseus, ammonium bicarbonate (ABC), sodium deoxycholate (SDC), and a mammalian total RNA extraction kit were purchased from Sigma-Aldrich (St. Louis, MO, USA). Peptide-*N*-glycosidase F (PNGase F) and 10 × G7 buffer (0.5 M sodium phosphate), NEBNext rRNA depletion kit (human/mouse/rat), and NEBNext Ultra II directional RNA library prep kit were obtained from New England Biolabs (Ipswich, MA, USA).

### 2.2. Preparation of Plasmax Medium

The Plasmax media was prepared according to the formulation and preparation steps provided by Voorde et al. [12]. Eight stock solutions were first prepared (see Table S1). For the components in solid phase, the required amount was weighed on an analytical balance, whereas the others in liquid phase were measured by volume using pipettes. For stock solution #1, all components were dissolved in 10 mL HPLC-grade water. Then, the pH value was adjusted to 1.00 using saturated HCl solution to increase the solubility of its components. The same method was applied to the preparation of stock solution #2, but no pH adjustment was needed due to the different composition from #1. For solution #3, the components were separated into two groups. The first group was prepared using the same method as for solutions #1 and #2 while the second group had the amount of the compositions multiplied by 10,000 and then dissolved in 1000 mL of HPLC-grade water. Finally, 9 mL of group 1 and 1 mL of group 2 were mixed, producing stock solution #3. Stock solution #4 only contained urate. The dissolution of the urate was achieved by adjusting the pH to 13.3 with concentrated NaOH followed by further sonication. Stock solution #5 contained BME vitamins and L-Glutamine, both of which are commercially available and in liquid phase, so they could be added into the final Plasmax medium directly without preparation. Solution #6 and solution #8 were prepared with the same method as solution #1. Solution #7 was Earle’s Balanced Salt Solution (EBSS), a base of Plasmax medium that was purchased directly from ThermoFisher Scientific. Stock solutions #1, 2, 3, 4, 6, and 8 were then stored in a −80 ℃ environment for future use.

Because of the large amount of nutrients contained in each stock solution, the mixing process needed to be performed in a strictly aseptic environment. To prepare a 500 mL bottle of Plasmax, first, the frozen stock solutions were thawed at room temperature, then 5 mL from both stock solutions #1 and #2 were added into 482 mL of EBSS. Then, 0.5 mL of solutions #3 and #8 were mixed. The volumes for solutions #4 and #6 were 1 and 0.05 mL, respectively. In total, 5 mL of BME-Vitamin, 1.625 mL of L-Glutamine, and 12.5 mL of fetal bovine serum (FBS) were also added. After adding all stock solutions and compositions, the mixture was shaken for 10 min to dissolve the components. To remove any bacteria that might be involved in the medium during the previous steps of preparation, a rapid-flow filter consisting of a 0.2 µm membrane was used. Finally, the fully prepared 500 mL Plasmax medium was stored in 4 ℃ for future use.

### 2.3. Cell Line Culturing and Harvesting

The cancer cell lines were cultured and harvested using previously reported procedures [26,27]. Briefly, MDA-MB-231BR and CRL-1620 were cultivated in Plasmax, DMEM, and EMEM, respectively. Initially, cells were cultured in 75 cm^2^ flasks, incubated at 37 °C for 4–8 days, and fed every 2–4 days. When the cell confluence reached 80%, cells were washed with a 10 mL aliquot of PBS twice and detached by 2.2 mL trypsin-EDTA solution. After incubation at 37 °C for 5 min, a 5 mL aliquot of fresh medium was added to the cell solution to neutralize trypsin. Next, the cell suspension solution was transferred to three 175 cm^2^ flasks for triplication and incubated at 37 °C for about 7 days, until 80% of cell confluence was reached. Cells were washed and detached using the method described above. After adding a 10 mL aliquot of the fresh medium, cells were harvested by centrifugation at 500× *g* for 5 min. Cell pellets were collected and washed twice with PBS to remove medium. Cell samples were stored in −20 °C until glycomics processing and analysis.

### 2.4. Protein Extraction

The proteins were extracted from cells using the procedures that were previously described [14,28,29]. Briefly, cells were thawed at room temperature. Then, 50 mM ammonium bicarbonate (ABC) buffer and 5% sodium deoxycholate (SDC) were added to resuspend the cells. The cell solution was transferred into vials with 400 µm zirconium beads for beads beating. To thoroughly break up the cells, each sample was shaken by a beads beater (BeadBug Microtube Homogenizer, Benchmark Scientific, Edison, NJ, USA) at 4 ℃ for 5 rounds with 30 s for each round and a 30 s interval to prevent overheating. Then, the samples were sonicated in ice water for 1 h. After that, the samples were centrifuged at 1000× *g* for 10 min and the supernatant was collected. Next, 2 µL solutions of each sample were taken for protein assay using a Micro BCA protein assay kit (Thermo Scientific/Pierce, Rockford, IL, USA). The final protein concentration was determined by a Multiskan plate reader (Thermo Scientific, Rockford, IL, USA).

### 2.5. Release and Permethylation of Glycans

After protein assay, *N*-glycans were released according to previously reported protocols [30,31]. Samples were initially denatured in a 90 ℃ water bath for 15 min. After cooling down to room temperature, 1 µL PNGase F was added to each sample, then incubated at 37 ℃ for 18 h. Then, formic acid (1% of sample volume) was added and vortexed to precipitate SDC. After centrifugation, supernatant was collected, and 90% ethanol was added and stored at −20 ℃ for 30 min to precipitate proteins. Then, the samples were centrifuged at maximum speed for 10 min. The supernatant was collected and dried. Next, samples were dissolved in 50 µL of water and dialyzed overnight against a 500–1000 MWCO dialysis membrane to remove salts and remaining SDC. After dialysis, samples were dried and reduced by 10 µL of borane-ammonia complex solution (10 mg/mL) at 60 ℃ for 1 h [32]. Reduced glycans were subjected to solid-phase permethylation [33,34,35,36,37]. Briefly, the reduced and dried glycans were suspended in 30 µL DMSO, 1.2 µL water, and 20 µL iodomethane. The spin columns were loaded with NaOH beads (suspended in DMSO) and spun down at 300× *g* for 2 min. Then, the columns were washed with 200 µL of DMSO. After the second centrifugation, the samples were transferred into the spin columns. After incubation for 25 min, another 20 µL of iodomethane was added to the spin columns and incubated for 15 min. Finally, the permethylated glycans were eluted by centrifuging the spin columns. The eluted glycans were dried overnight and resuspended in an aqueous solution containing 20% acetonitrile and 0.1% formic acid, prior to LC-MS/MS analysis.

For *O*-linked glycans, an enzymatic/chemical method was utilized to efficiently release any permethylated *O*-glycans [38]. Following the protein extraction and protein assay parts as described above, the extracted protein samples were treated with pronase at 37 ℃ for 48 h. Then, formic acid (1% of sample volume) was added to remove SDC. The samples were dialyzed (50–100 MWCO) overnight to remove salts, followed by drying in the speed vacuum. Finally, the samples were subjected to solid-phase permethylation as described above. Due to the basic condition, *O*-glycans were released and permethylated during the permethylation process.

### 2.6. Total RNA Extraction and Transcriptomics Analysis

To investigate the glycan expression changes in different media at the transcriptomics level, total RNAs were extracted from both cell lines using a mammalian total RNA extraction kit following the vendor’s procedures. Biological triplicates were prepared and 5 μL of total RNA solution from each sample was taken for concentration measurement using a Nanodrop ND-1000 spectrophotometer (Thermo Scientific, Wilmington, DE, USA). Then, RNA quality was determined using RNA Screen Tape (Agilent Technologies, Santa Clara, CA, USA). Ribosomal RNA depletion was performed using a NEBNext rRNA depletion kit (human/mouse/rat). RNA fragmentation, double-stranded cDNA, and adaptor ligation were generated using a NEBNext Ultra II directional RNA library prep kit. PCR enriched libraries were quantified by Qubit and equimolar indexed libraries were pooled. Pooled libraries were quantitatively checked using software 2200 Tapestation (Agilent Technologies, Santa Clara, CA, USA). Next, the libraries were diluted to 200 pM and spiked with 2% phiX libraries (Illumina control). The transcriptome sequencing was performed on the barcoded stranded RNA-Seq libraries using an Illumina NovaSeq 6000 S1 flow cell (Illumina, San Diego, CA, USA) and paired-end reads (2 × 100 bp).

### 2.7. LC-MS/MS Glycomics Analysis

The glycan samples were analyzed by a Dionex UltiMate 3000 nano LC system (Dionex, Sunnyvale, CA, USA) coupled with an LTQ Orbitrap Velos mass spectrometer (Thermo Scientific, San Jose, CA, USA) through a nano-ESI source. A C18 Acclaim PepMap 100 trapping column (75 μm I.D. × 2 cm, 3 μm particle sizes, 100 Å pore sizes, Thermo Scientific, San Jose, CA, USA) was used for online purification. The glycan separation was achieved by an Acclaim C18 nano column (75 μm I.D. × 15 cm, 2 μm particle sizes, 100 Å pore sizes, Thermo Scientific, San Jose, CA, USA), with the flow rate set at 350 nL/min. The column temperature was maintained at 55 ℃ during the separation. Mobile phase A was an aqueous solution that contained 2% of acetonitrile and 0.1% of formic acid, while mobile phase B was acetonitrile with 0.1% formic acid. An optimized 60 min LC method was applied with an elution gradient that started with 20% of solvent B for the first 10 min, then increased to 42% in 1 min, 42% to 55% in 37 min, and 55% to 90% in 1 min. After holding at 90% for 5 min, the gradient was dropped to 20% in 1 min and held for 5 min to rebalance the column. The LTQ Orbitrap Velos mass spectrometer was operated in positive mode for the full MS scan (1st scan event) with the *m*/*z* range set at 700–2000 and 400–2000 for *N*- and *O*-glycan detection, respectively. The mass resolution was 100,000. Then, the top 4 intense ions were selected for CID (collision-induced dissociation) fragmentation (2nd scan event), with a collision energy of 35% and activation time of 10 ms. The injection amount of each sample was normalized by the protein amount. Based on the protein assay results, all samples were resuspended to the same final concentration. For each sample, the glycans released from 50 μg of proteins were injected into LC-MS/MS for identification and quantitation.

### 2.8. Data Analysis

After analyzing the *N*- and *O*-glycans of the two cancer cell lines cultivated in the three different media using LC-MS/MS, the raw data files of the glycans were processed with an in-house software, MultiGlycan [39], with 6 ppm mass tolerance to identify glycans. The glycan structures identified by MultiGlycan were validated by a manual check using Xcalibur (Thermo Scientific, v4.4) to remove the false positives. In Xcalibur, a 10 ppm mass tolerance in full MS was set to search the *m*/*z* of the corresponding glycan structures. Then, the structures were manually confirmed by checking the full MS and MS^2^. The absolute abundance of each glycan was calculated by adding the intensities of all adduct forms and charge states together. Then, the glycan abundance was normalized by the relative abundance, which was achieved by dividing the individual glycan abundance by the total glycome abundance. Then, the relative abundance of all glycans from each cell line in different media were subjected to unsupervised principal component analysis (PCA) using MarkerView software (Sciex, v1.3) for an overall comparison of differentially expressed glycans. Both *N*- and *O*-glycans were categorized according to their monosaccharide compositions to study the expression changes of different types of glycans. Two-tailed student *t* tests were employed to identify the statistically significant glycans of the cell lines cultivated in Plasmax and the two commercial media.

## 3. Results

### 3.1. Culturing Media Specification

The concentrations of the nutrients and metabolites of the Plasmax media were based on the freely available resource (www.serummetabolome.ca, accessed on 11 January 2019) [40]. The direct comparison of the formulation of Plasmax and the other two commercial media is shown in Supporting Information Figure S1 and Table S2. Glucose and glutamine are approximately 60% of all nutrients in EMEM and more than 75% in DMEM; however, these two nutrients are only 43% in Plasmax. Furthermore, 35% of the total nutrients and metabolites in Plasmax are absent in the two commercial media, including urea, lactate, and urate. In addition, some trace elements, such as zinc and manganese, are only present in Plasmax.

### 3.2. N-Glycan Identification

Since the positional isomers are very common in *N*-glycans, such as core- and branch-fucose, the process of *N*-glycan identification in this study included two major steps. First, the monosaccharide composition was confirmed using full MS with a mass tolerance less than 5 ppm. Second, the MS/MS spectra were generated for the structural identification by searching for diagnostic fragment ions. Supporting Information Figure S2A shows an example from the 231BR cell line. Based on the theoretical *m*/*z* value of [M + 2H]^2+^ = 915.4908, the EIC was extracted, with the full MS spectrum depicted by the inset. The composition was confirmed as HexNAc_4_Hex_3_DeoxyHex_1_, which is a potential fucose positional isomer. Then, the MS/MS spectrum in Figure S2B was utilized to elucidate the structure. The peak of *m*/*z* = 468.3 ([M + H]^+^) represents the diagnostic fragment ion of core-fucosylation. It contains one fucose connected with one GlcNAc on the reducing end, which is the unique part of core-fucosylataed *N*-glycans. Therefore, this structure of HexNAc_4_Hex_3_DeoxyHex_1_ was confirmed as core-fucosylataed. Another example from the CRL cell line is shown in Figure S2C,D. The composition of HexNAc_4_Hex_5_DeoxyHex_1_ (*m*/*z* = 1119.5905, [M + 2H]^2+^) was confirmed through full MS. The MS/MS spectra assigned the fucose to the core GlcNAc as the diagnostic ion of *m*/*z* = 468.3 was also observed for this structure. By applying the same strategy, more than 50 unique *N*-glycan structures were identified from each cell line cultivated in three media. Core-fucosylation was mostly found among the fucosylated *N*-glycans derived from the two types of cancer cell lines used for this study.

### 3.3. Chemometric Analysis of N-Glycans by Unsupervised Principal Component Analysis

**Figure 1:**
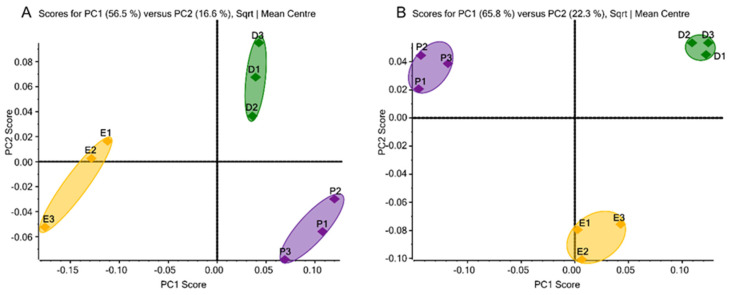
Unsupervised principal component analysis of *N*-glycans derived from (**A**) the 231BR cell line, with 49 glycans, 48 glycans, and 50 glycans identified in DMEM, EMEM, and Plasmax, respectively, and (**B**) the CRL cell line, with 50 glycans identified in each of the three media. Symbols: ◆, DMEM; ◆, EMEM; ◆, Plasmax.

Overall, a total of 52 *N*-glycans from the 231BR cell line and 51 *N*-glycans from the CRL cell line were identified (Supporting Information Tables S3A and S4A, respectively) and subjected to PCA. As a chemometric approach to simplify high-dimensional data sets into lower-dimensional sets, PCA uses the orthogonal transformation to convert possibly correlated variables into principal components to minimize information loss. It displays the similarities and differences of data groups by plotting in a map [41]. Figure 1A,B depicts the unsupervised PCA plots of quantitative glycomics data from the 231BR and CRL cell lines, respectively, cultivated in three media. The closely located triplicate results of PCA suggest the well-reproduced and reliable quality of our glycomic analyses. Complete separation of the three different clusters can be observed through the primary principal component (PC1) and secondary principal component (PC2), which indicates the differences in the glycan expressions in the three media. In 231BR, the *N*-glycan expressions in DMEM and Plasmax were different in the PC2 score, while EMEM was different from both DMEM and Plasmax in the PC1 score. However, in CRL, DMEM and Plasmax were found to be different in the PC1 score, and both were different from EMEM in terms of PC2.

### 3.4. N-Glycan Expression Changes among Plasmax, DMEM, and EMEM

**Figure 2:**
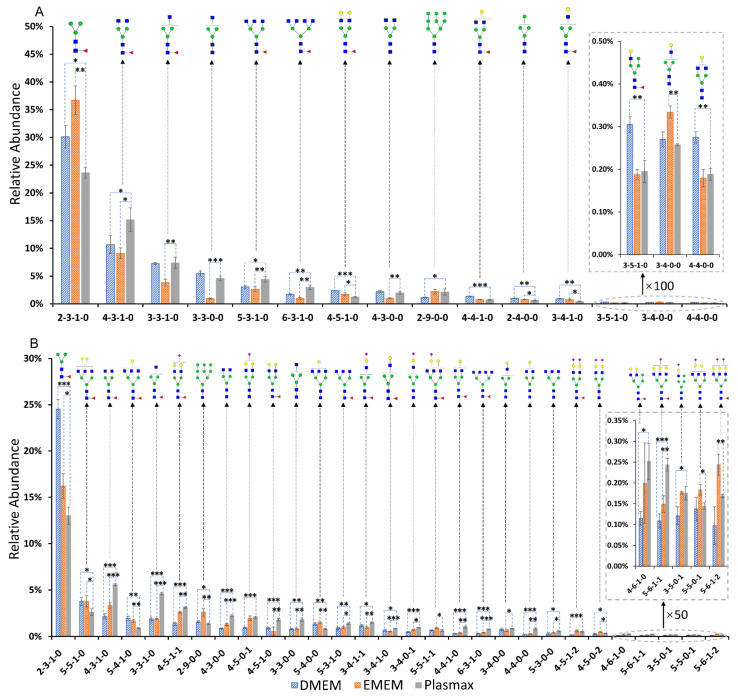
Relative abundance of significant *N*-glycan structures from (**A**) 231BR and (**B**) CRL cell lines. Error bars denote the standard deviation from three biological replicates. The glycan composition is given by a 4-digit code. The first number denotes the number of *N*-acetylglucosamine (HexNAc); the second number denotes the number of hexose (Hex); the third number denotes the number of fucose (DeoxyHex); and the fourth number stands for the number of sialic acid (NeuAc). Symbols: 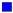, N-acetylglucosamine (GlcNAc); 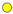, Galactose (Gal); 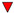, Fucose (Fuc); 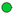, Mannose (Man); 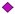, N-acetylneuraminic acid (NeuAc/Sialic Acid). *: *p*-value < 0.05; **: *p*-value < 0.005; ***: *p*-value < 0.0005.

The comparisons of the *N*-glycan expressions in Plasmax and commercial media were performed for both cancer cell lines. Student’s *t*-test was utilized for the statistical test. In the breast cancer cell line 231BR, 15 *N*-glycans were found with significant changes (*p* < 0.05) in the relative abundance when cultivated in Plasmax compared to DMEM and EMEM. On the other hand, 29 *N*-glycans, derived from the brain cancer cell line CRL, showed significantly different expressions between Plasmax and the two commercial media. Bar graphs (Figure 2) of the significant *N*-glycans from the two cell lines were generated to assess the up- or downregulation of the relative abundance of each structure. The asterisks denote the significant level of each glycan in Plasmax compared to the commercial media. The majority of *N*-glycans that exhibited significant expression changes were neutral structures with or without fucosylation. For the 231BR cell line, the significant *N*-glycans include six other structures and nine fucosylated structures. Furthermore, multiple fucosylated *N*-glycans with GlcNAc at the end of the branches (HexNAc_4_Hex_3_DeoxyHex_1_, HexNAc_5_Hex_3_DeoxyHex_1_ and HexNAc_6_Hex_3_DeoxyHex_1_) were upregulated in Plasmax. Interestingly, the expressions of the fucosylated structures with galactose at the end of the branches tended to be more active in DMEM (see HexNAc_4_Hex_5_DeoxyHex_1_, HexNAc_4_Hex_4_DeoxyHex_1_, and HexNAc_3_Hex_4_DeoxyHex_1_ in Figure 2A).

While, in CRL cell line, 7 other and 11 fucosylated structures exhibited significant changes in terms of the relative abundances. Similar to 231BR, upregulation of HexNAc_4_Hex_3_DeoxyHex_1_, HexNAc_5_Hex_3_DeoxyHex_1_, and HexNAc_6_Hex_3_DeoxyHex_1_ was also observed. Furthermore, the relative abundance of HexNAc_3_Hex_3_DeoxyHex_1_ was doubled by Plasmax in relation to DMEM and EMEM. Some sialo-fucosylated structures (*N*-glycans contain both sialic acid and fucose) were also observed, with a higher abundance in Plasmax, including HexNAc_4_Hex_5_DeoxyHex_1_NeuAc_1_, HexNAc_3_Hex_4_DeoxyHex_1_NeuAc_1_, and HexNAc_5_Hex_6_DeoxyHex_1_NeuAc_1_. In addition, significant differences were also found among sialylated structures from the CRL cell line, such as HexNAc_3_Hex_5_NeuAc_1_, HexNAc_5_Hex_5_NeuAc_1_, and HexNAc_5_Hex_6_NeuAc_2_ (inset of Figure 2B). These *N*-glycans were observed with the highest and the lowest relative abundance in EMEM and DMEM, respectively.

The comparison results of the different expressions of these significant *N*-glycans are also shown in Supporting Information Figure S3, which illustrates the up- or downregulation using heat maps. The first three columns depict the triplicates of the relative abundance of *N*-glycans from cell lines cultivated by DMEM, the second three columns are triplicates cultivated by EMEM, and the last three columns were cultivated by Plasmax. The red color denotes upregulation, while the green color denotes downregulation. Overall, among these significant *N*-glycans in the 231BR cell line, seven *N*-glycans were upregulated by Plasmax compared to the commercial media, while eight *N*-glycans were downregulated. In the CRL cell line, 20 *N*-glycans were upregulated and 9 *N*-glycans were downregulated.

**Figure 3:**
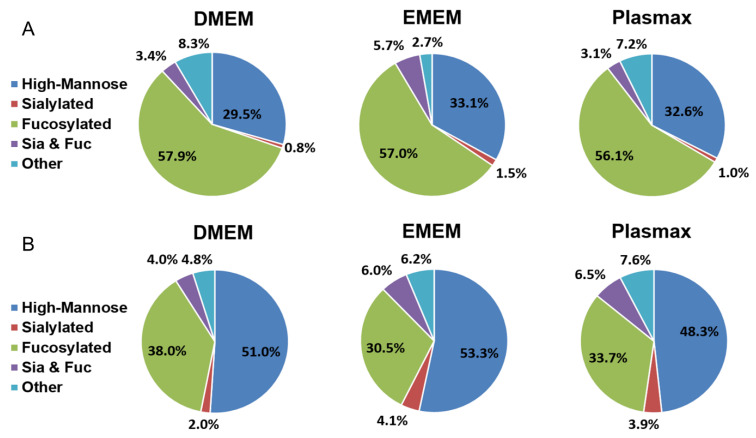
Distribution comparison of different types of *N*-glycans derived from (**A**) the 231BR cell line and (**B**) CRL cell line cultivated in DMEM, EMEM, and Plasmax.

Based on the monosaccharide compositions, all identified *N*-glycans were classified into five categories, including high mannose, sialylated, fucosylated, sialo-fucosylated, and other structures. The mean of the relative abundance of each *N*-glycan type was calculated (*n* = 3) and the pie charts in Figure 3 were generated. Student’s *t*-test was applied to assess the statistical significance between each two media (Table 1). The distribution of *N*-glycan types in 231BR cells is demonstrated in Figure 3A. The relative abundances of all fucosylated and high mannose structures were comparable between the three media. DMEM exhibited a fucosylated level of 57.9%, while in EMEM and Plasmax, 57% and 56.1% of *N*-glycans were fucosylated, respectively. In Plasmax, 32.6% of the *N*-glycans were high mannose structures, which is 3.1% higher than in DMEM but 0.5% lower than in EMEM. Other structures accounted for 8.3% and 7.2% in DMEM and Plasmax, respectively, which are both significantly higher than the 2.7% in EMEM (*p* = 2 × 10^−4^). A relatively lower amount of sialylated glycans was observed in all three media, with 0.8% in DMEM, 1.5% in EMEM, and 1.0% in Plasmax. Sialo-fucosylated structures also had the highest abundance of 5.7% in EMEM, relative to 3.4% and 3.1% in DMEM and Plasmax, respectively.

Figure 3B demonstrates the distribution of different types of *N*-glycans from the CRL cell line. High-mannose *N*-glycans showed the highest abundance in all three media. Both DMEM and EMEM have more than 50% of their *N*-glycans as high mannose, while the 48.3% in Plasmax is significantly lower than the 53.3% in EMEM (Table 1, *p* = 3 × 10^−4^). The amounts of sialylated *N*-glycans were relatively higher compared to the 231BR cell line. A total of 3.9% and 4.1% sialylated structures in Plasmax and EMEM, respectively, is both significantly higher than the 2.0% in DMEM (*p* = 1 × 10^−3^). The highest fucosylation level was observed in DMEM at 38.0%, which is 4.3% higher than Plasmax (*p* = 0.04). In EMEM, however, the abundance of fucosylated *N*-glycans was 3.2% lower than that in Plasmax (*p* = 0.04). Sialo-fucosylated *N*-glycans showed the highest abundance in Plasmax of 6.5%, which is upregulated by 2.5% relative to DMEM (*p* = 9 × 10^−4^). Other structures accounted for 7.6% in Plasmax, which is significantly higher than the 6.2% in EMEM (*p* = 0.02) and 4.8% in DMEM (*p* = 1 × 10^−3^).

### 3.5. Chemometric Analysis of O-Glycans by Unsupervised Principal Component Analysis

For *O*-glycans, the same data processing strategies were applied. First, we identified the *O*-glycan structures in two cancer cell lines cultivated by three media using the software MultiGlycan and manually checked the results using Xcalibur. A total of 27 and 31 *O*-glycans were identified and quantified from the 231BR and CRL cell lines, respectively (Supporting Information Tables S3B and S4B). Then, all identified *O*-glycans from each cell line were subjected to unsupervised PCA for an overall comparison of the *O*-glycan expressions in the three different media.

**Figure 4:**
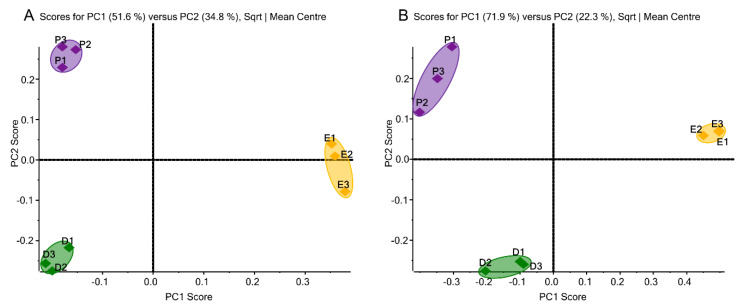
Unsupervised principal component analysis of *O*-glycans derived from (**A**) the 231BR cell line, with 20 glycans, 9 glycans, and 22 glycans identified in DMEM, EMEM, and Plasmax, respectively, and (**B**) the CRL cell line, with 28 glycans, 18 glycans, and 27 glycans identified in DMEM, EMEM, and Plasmax, respectively. Symbols are the same as in Figure 1.

As shown in Figure 4, for both the 231BR and CRL cell line, the three replicates of each media are well-grouped and each group is separated from each other, which suggests the high reproducibility between the replicates and the discrepancies in the *O*-glycan expressions among different media, respectively. In addition, the differences in the *O*-glycans expressions in the three media were found to be similar between the 231BR and CRL cell lines: Both triplicates of Plasmax showed higher PC2 scores than DMEM, while EMEM was different from both Plasmax and DMEM in terms of the PC1 score. Furthermore, EMEM has the least number of unique *O*-glycans identified, while DMEM and Plasmax have a comparable number of confirmed *O*-glycans.

### 3.6. O-Glycan Expression Changes among Plasmax, DMEM, and EMEM

**Figure 5:**
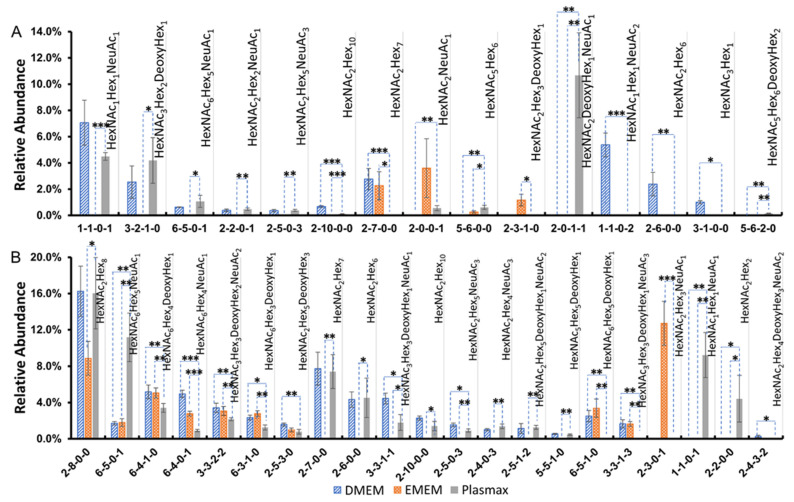
Relative abundance of significant *O*-glycans derived from (**A**) the 231BR cell line and (**B**) CRL cell line. The four-digit number labeled on the x-axis represents the structure of *O*-glycans. The first number represents HexNAc, the second represents Hex, the third represents fucose (DeoxyHex), and the last represents NeuAc/sialic acid. Each bar represents the mean of three biological replicates; the error bars denote the standard deviation. *: *p*-value < 0.05; **: *p*-value < 0.005; ***: *p*-value < 0.0005.

By comparing the relative abundance of each identified *O*-glycan in different media, 15 structures from the 231BR cell line and 21 structures from the CRL cell line exhibited significant expression changes (*p* < 0.05). Figure 5 depicts the relative abundance of significant *O*-glycans in both cell lines. Each *O*-glycan is labeled by monosaccharide compositions, which were confirmed based on their *m*/*z* values. Figure 5A gives the results of the 231BR cell line. Among these 15 significant *O*-glycan structures, only 4 structures were detected in EMEM, including HexNAc_2_Hex_7_, HexNAc_2_NeuAc_1_, HexNAc_5_Hex_6,_ and HexNAc_2_Hex_3_DeoxyHex_1_. In addition, the sialo-fucosylated structure HexNAc_2_DeoxyHex_1_NeuAc_1_ showed the highest relative abundance when cultured in Plasmax. However, this *O*-glycan was not detected in DMEM and EMEM, indicating that the nutritional environment of Plasmax might be more suitable for the expression of this glycan. Furthermore, HexNAc_1_Hex_1_NeuAc_2_, HexNAc_2_Hex_6_, and HexNAc_3_Hex_1_ were exclusively detected in DMEM. The abundances of fucosylated structures such as HexNAc_3_Hex_2_DeoxyHex_1_ and HexNAc_5_Hex_6_DeoxyHex_2_ were higher in Plasmax. The neutral structure HexNAc_2_Hex_10_ showed a higher abundance in DMEM compared to Plasmax.

Figure 5B demonstrates the 21 significant *O*-glycans of the CRL cell line. Similar to the results we found in the 231BR cell line, multiple *O*-glycans of CRL were absent in EMEM, suggesting that the nutritional environment of EMEM might not be very supportive of *O*-glycan expressions in cancer cell lines. Therefore, we focused on the expression differences between DMEM and Plasmax. Sialylated structures such as HexNAc_6_Hex_5_NeuAc_1_ and HexNAc_1_Hex_1_NeuAc_1_ exhibited significantly higher abundances in Plasmax (*p* < 0.005). However, four fucosylated structures, including HexNAc_6_Hex_4_DeoxyHex_1_, HexNAc_6_Hex_3_DeoxyHex_1_, HexNAc_2_Hex_5_DeoxyHex_3_, and HexNAc_6_Hex_5_DeoxyHex_1_, had lower relative abundances in Plasmax when compared to DMEM. In addition, another four sialo-fucosylated *O*-glycans, including HexNAc_3_Hex_3_DeoxyHex_2_NeuAc_2_, HexNAc_3_Hex_3_DeoxyHex_1_NeuAc_1_, HexNAc_3_Hex_3_DeoxyHex_1_NeuAc_3_, and HexNAc_2_Hex_4_DeoxyHex_3_NeuAc_2_, also had lower level of relative abundance in Plasmax than in DMEM. Other *O*-glycan structures, such as HexNAc_2_Hex_6_ HexNAc_2_Hex_7_ and HexNAc_2_Hex_8_, showed comparable abundances between Plasmax and DMEM. As reported by the previous study [38], none of the *N*-glycans were detected after applying the same method to release *O*-glycan, thus we have a high confidence in identifying these structures as *O*-glycans. However, since the sample species were different in this study, we cannot completely exclude the possibility of *N*-glycans. This needs to be investigated in future studies. The significant up- and downregulation of *O*-glycans in the three media was also compared and is shown in heat maps in Supporting Information Figure S4. The 231BR cell line has seven upregulated *O*-glycans and eight downregulated *O*-glycans in Plasmax. However, CRL cell line has 12 upregulated *O*-glycans and 9 downregulated *O*-glycans.

**Figure 6:**
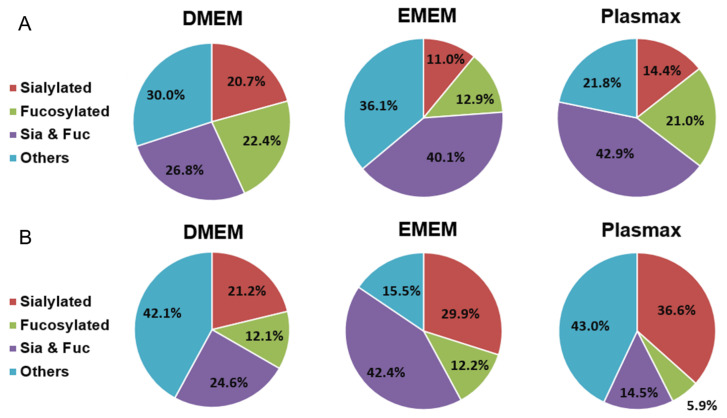
Distribution comparison of different types of *O*-glycans derived from (**A**) the 231BR cell line and (**B**) CRL cell line cultivated in DMEM, EMEM, and Plasmax.

To study the global effects of different media on the expressions of *O*-glycans, the identified structures were grouped into four different types, including sialylated, fucosylated, sialo-fucosylated, and other structures. As shown in Figure 6, the distributions of the four types of *O*-glycans from both cell lines cultivated in three media are presented by the pie charts, with Table 2 demonstrating the differences between each two media. In the 231BR cell line, sialo-fucosylated structures accounted for 42.9% in Plasmax, which is significantly higher than the 26.6% in DMEM (*p* = 0.01). Fucosylated structures, with 22.4% in DMEM and 21.0% in Plasmax, were both higher than the 12.9% in EMEM. The highest sialylation level was observed in DMEM, which is 6.3% higher than Plasmax (*p* = 4 × 10^−3^). For other structures, no significant difference was observed between the media.

For the CRL cell line, the different distributions of *O*-glycans are shown in Figure 6B. Both DMEM and Plasmax showed more than 40% of other structures. In addition, DMEM and EMEM had significantly higher abundances of sialo-fucosylated structures than Plasmax (*p* = 0.02 and *p* = 1 × 10^−4^, respectively). On the other hand, CRL cells cultured in Plasmax exhibited the highest expression level of sialylated *O*-glycans, which is 15.4% higher than DMEM (*p* = 0.01). In contrast, the fucosylation level in Plasmax was the lowest when compared to that in DMEM (*p* = 4 × 10^−3^) and EMEM (*p* = 5 × 10^−5^).

### 3.7. Expression Changes of Glycogenes in Plasmax

Glycotransferase plays an important role during the synthesis processes of *N*- and *O*-glycans; therefore, the transcriptomics analysis of glycosylation genes can provide deeper understandings of the alterations in Plasmax. In our study, we focused on the glycogenes that are associated with the glycan expression changes described above. All detected glycogenes are shown in Supporting Information Table S5. For the 231BR cell line, Supporting Information Figure S5A depicts the transcript expressions of multiple galactosyltransferases (B4GALTs) in terms of RPKM, among which, B4GALT1 was 3.07-fold upregulated in DMEM compared to Plasmax, which can induce more galactosylation of glycans [42]. Figure S5B provides information of the *N*-acetylglucosaminyltransferase (MGAT) transcripts. Both MGAT1 and MGAT2 were more than 2-fold upregulated in Plasmax over EMEM, leading to activated GlcNAc addition and branching of *N*-glycans [43]. Figure S5C,D illustrates the synthesis pathways of complex glycans, and branching of *N*-glycans, respectively. The related transcripts of glycotransferases are labeled. Similar changes in MGATs were also observed in CRL cell lines as shown by Figure S6A, which are correlated with multiple upregulated neutral *N*-glycans in Plasmax. In addition to that, a higher abundance of fucosyltransferases was also observed in Plasmax compared to EMEM (Figure S6B). Specifically, core-fucosyltransferase (FUT8) was 1.8-fold upregulated in Plasmax. These transferases were also highlighted in the synthesis pathways shown in Figure S6C. On the other hand, compared to DMEM, lower abundances of sialylated and fucosylated *O*-glycans were observed in the 231BR and CRL cell lines, respectively. The correlated transcripts of sialyltransferases and fucosyltransferases were also identified as shown in Figure S7. For example, α-1,3-fucosyltransferase 7 (FUT7), which participates in the biosynthesis of the sialyl Lewix X [44], was decreased by 18% in Plasmax relative to DMEM. Another fucosyltransferase, FUT9, has also been reported to be involved in the expression of Lewis X [45]. However, probably due to the low abundance, it was not detected in our transcriptomics analysis. Although the abundance changes of the abovementioned transcripts of glycotransferases were not statistically significant (*p* > 0.05) in different media, we observed obvious fold changes, which might contribute to the subsequent glycomics biosynthesis enzymes, thus regulating the glycan synthesis processes.

## 4. Discussion

Cell line cultivation is a vital part of in vitro studies of disease marker candidate discovery, which is also one of the main strategies of current cancer research. As a major material for the experiment, the culturing media should be carefully taken into consideration to provide a suitable nutritional environment for cell lines, since it can determine the proliferation, transcriptional, and metabolic phenotypes of cells. The novel physiological medium, Plasmax, composed of nutrients and metabolites found in human plasma, has been reported to enhance colony formation and alter the metabolism of cells. Moreover, Plasmax can closely mimic the in vivo environment of tumors compared with the commercial medium DMEM-F12 [12]. However, an investigation of the glycan expressions of cancer cell lines using Plasmax as the culturing media has not yet been conducted. Compared to healthy cells, glycosylation changes in cancer cells have been discovered [46], leading to research on glycan biomarkers to facilitate clinical diagnostics and therapeutics [47,48]. Moreover, it has been reported that protein glycosylation differences exist between DMEM-cultured colorectal cancer cell lines and epithelial cells from colorectal tumor tissue [49]. Considering the different environments for cancer cell growth, the culturing media could be one of the main reasons that discrepancies were introduced. To study the influence of Plasmax on glycan expressions, we performed *N*- and *O*-glycomes profiling of cancer cell lines cultivated in Plasmax, and compared the results with the expression results in commercial media DMEM and EMEM. Furthermore, the transcriptomics of cells cultured in different media was also investigated, focusing on glycosylation gene expression. These glycosylation gene expressions were correlated with glycomic changes to acquire a deeper insight into glycosylation alterations in Plasmax.

After the cell harvest and protein extraction, the amount of proteins in each sample was normalized utilizing a BCA protein assay. For *N*-glycan analysis, each run contained reduced and permethylated glycans after enzymatical release from 100 μg of proteins using PNGase F. On the other hand, *O*-glycans released from 300 μg of proteins were injected for each analysis. Unlike *N*-glycans, so far, there are fewer efficient and reliable enzymes for the cleavage of *O*-glycans from glycoproteins; therefore, chemical release methods such as *β*-elimination are frequently used for *O*-glycomics studies [50,51]. However, samples that are subjected to conventional *β*-elimination can suffer from significant sample loss during the cleaning step for the removal of extra salt. In our work, we followed an enzymatic/chemical approach, which minimizes the sample loss and improves the sensitivity of detection. Moreover, this method does not lead to detectable co-release of *N*-glycans according to the reported results [38]. Starting with nonspecific pronase digestion of proteins, followed by solid-phase permethylation, the high alkaline conditions initiate the *β*-elimination process, thus releasing *O*-linked glycans. The following permethylation reaction converts the released *O*-glycans to their permethylated derivatives, which improves the structural stability and the ionization efficiency of glycans during the electrospray ionization (ESI) process in positive ion mode [52,53]. Moreover, permethylated glycans possess higher hydrophobicity, which boosts the affinity between glycans and the reversed-phase LC column; therefore, the separation performance is increased. The LC system was equipped with a C18 trapping column for online purification to remove impurities and salt and further increased the ionization efficiency of glycans. The high sensitivity and mass accuracy of the LTQ Orbitrap Velos mass spectrometer enabled the detection of low-abundant glycans.

A total of 52 and 51 unique *N*-glycan structures were identified and quantified in the 231BR and CRL cell lines, respectively. The relative abundances of the glycans were calculated and subjected to unsupervised PCA. The clusters of the triplicates cultivated in Plasmax were clearly separated from the commercial media in both cell lines, indicating the significantly different glycan expressions in this physiological media. In addition, the DMEM and EMEM clusters were also separate from each other, which could be caused by the higher concentrations of certain nutrients in DMEM, although the compositions of both commercial media are very similar. Moreover, the PCA plots from the two cancer cell lines exhibited different distributions of the three media in terms of the PC1 and PC2 scores as described above, suggesting that the influence of Plasmax on *N*-glycosylation is cell line specific. For *O*-glycans, overall, 27 and 31 unique structures were observed and studied in the 231BR and CRL cell lines, respectively. Regarding the *O*-glycan expressions in each media, the amounts of *O*-glycans identified in EMEM were relatively lower compared to Plasmax and DMEM. For both cell lines, around 10 structures identified in the latter two media were absent in EMEM, suggesting that EMEM might not be able to provide a suitable environment for the expression of *O*-glycomes. The limited *O*-glycan expression in EMEM is also the main reason for the different PC1 scores compared to Plasmax and DMEM shown in the PCA plots.

The glycans with a significantly different relative abundance (*p* < 0.05) were investigated between Plasmax and the two commercial media. The differentially expressed 15 *N*-glycans of the 231BR cell line are all neutral structures without sialic acid, suggesting that Plasmax does not have a perceptible influence on the sialylation of *N*-glycans in this type of cancer cell line. According to the distribution of different types of *N*-glycans, no significant differences were observed regarding fucosylation. However, significantly higher abundances of fucosylated structures HexNAc_4_Hex_3_DeoxyHex_1_, HexNAc_5_Hex_3_DeoxyHex_1_, and HexNAc_6_Hex_3_DeoxyHex_1_ were observed in Plasmax than both DMEM and EMEM. In addition, the other fucosylated structures, such as HexNAc_4_Hex_5_DeoxyHex_1_, HexNAc_4_Hex_4_DeoxyHex_1_, HexNAc_3_Hex_5_DeoxyHex_1_, and HexNAc_3_Hex_4_DeoxyHex_1_, have lower relative abundances, which draws our attention to the galactose on the *N*-glycan branches. The overexpression of galactosylated *N*-glycans in commercial media, especially in DMEM, is associated with the upregulation of B4GALTs in the Golgi apparatus. For the fucosylated HexNAc_4_Hex_3_DeoxyHex_1_ and its non-fucosylated counterpart HexNAc_4_Hex_3_, both structures exhibited the lowest abundance in EMEM; similar results were also observed in another pair of *N*-glycans: HexNAc_3_Hex_3_DeoxyHex_1_ and HexNAc_3_Hex_3_. If we take the most abundant significant *N*-glycan HexNAc_2_Hex_3_DeoxyHex_1_ into consideration, we observe a complementary distribution of the relative abundance of this structure in the three media. Such results indicate a lower chance of adding *N*-acetylglucosamine (GlcNAc) onto the mannose of the *N*-glycan core structure when cultivating in EMEM, which also agrees with the pie charts, where the lowest abundance of other structures was identified in EMEM among the three media. The addition of GlcNAc in *N*-glycans is catalyzed by the enzyme *N*-acetylglucosaminyltransferase (MGAT) in the Golgi, and this process is predicted to be inhibited in the 231BR cell line cultivated by EMEM according to our transcriptomics results. Furthermore, the upregulation of tri-antennary HexNAc_5_Hex_3_DeoxyHex_1_ and tetra-antennary HexNAc_6_Hex_3_DeoxyHex_1_ in Plasmax suggest that GlcNAc addition and the branching processes of *N*-glycans are more active than both commercial media.

In contrast to the 231BR cell line, a total of 29 *N*-glycans derived from the CRL cell line were listed as significant, suggesting more remarkable changes in glycosylation in this cell line. The more activated GlcNAc addition of *N*-glycans in Plasmax was also observed in the CRL cell line, including HexNAc_4_Hex_3_, HexNAc_3_Hex_3_DeoxyHex_1_, HexNAc_3_Hex_3_, HexNAc_5_Hex_3_DeoxyHex_1_, and HexNAc_6_Hex_3_DeoxyHex_1_, which agrees with the transcriptomics expression changes in *N*-acetylglucosaminyltransferase. Meanwhile, HexNAc_2_Hex_3_DeoxyHex_1_ still showed the lowest abundance in Plasmax. On the other hand, DMEM exhibited the lowest level of sialylation in the CRL cell line, which is correlated with the catalyzation of sialyltransferases that takes place in the Golgi apparatus [54]. This process is predicted to be more active in Plasmax, based on the upregulated HexNAc_4_Hex_5_NeuAc_1_, HexNAc_3_Hex_4_NeuAc_1_, and HexNAc_3_Hex_5_NeuAc_1_. Moreover, the transcriptomics data suggests that the α2, 6-sialyltransferase (ST6GAL2), was only detected in Plasmax. The fucosylated *N*-glycans in the CRL cell line showed a higher abundance in Plasmax than EMEM, which indicates increased stimulated fucosylation in the CRL cell line when cultivated in Plasmax. Combining the sialylation and fucosylation, several *N*-glycans with both sialic acid and fucose were found to be overexpressed in Plasmax, including HexNAc_4_Hex_5_DeoxyHex_1_NeuAc_1_, HexNAc_3_Hex_4_DeoxyHex_1_NeuAc_1_, and HexNAc_5_Hex_6_DeoxyHex_1_NeuAc_1_. These changes are driven by the combination of more activated sialyltransferases and fucosyltransferases in Plasmax compared with DMEM and EMEM.

Due to the fewer *O*-glycans identified in both cell lines cultivated by EMEM, we do not recommend the use of EMEM as the culturing medium for *O*-glycomics studies of cell lines as it might limit the *O*-glycan expression or *O*-glycosylation. The comparisons between Plasmax and DMEM will mainly be discussed. For the 231BR cell line, a significant decrease in sialylated structures was observed in Plasmax. A good example is HexNAc_1_Hex_1_NeuAc_2_, which was only detected in DMEM. According to the transcriptomics results, several sialyltransferases were upregulated in DMEM, including both α2, 3- and α2, and 6-sialyltransferases (ST3GAL and ST6GAL), which stimulated the corresponding processes [55]. On the other hand, the sialo-fucosylated *O*-glycan HexNAc_2_DeoxyHex_1_NeuAc_1_ was solely found in Plasmax. Since the fucosylation levels in Plasmax and DMEM are comparable, there is not enough evidence to correlate the expression change of HexNAc_2_DeoxyHex_1_NeuAc_1_ with fucosyltransferases that are involved in *O*-glycan expression. Moreover, because of the various core structures of *O*-glycans and the complex enzymatic activities that occur during the modification of sialo-fucosylated *O*-glycans, further study is required to build the connections between the expression changes of this type of *O*-glycan with enzymes in the 231BR cell line. On the other hand, the sialylation of *O*-glycans in the CRL cell line was increased in Plasmax (such as HexNAc_1_Hex_1_NeuAc_1_ and HexNAc_6_Hex_5_NeuAc_1_), while fucosylation was decreased compared to DMEM (HexNAc_6_Hex_4_DeoxyHex_1_, HexNAc_6_Hex_3_DeoxyHex_1_, and HexNAc_2_Hex_5_DeoxyHex_3_). Both changes are similar to those found in *N*-glycans of CRL. However, in contrast to *N*-glycans, the sialo-fucosylated *O*-glycans showed a lower abundance in Plasmax, such as HexNAc_3_Hex_3_DeoxyHex_2_NeuAc_2_, HexNAc_3_Hex_3_DeoxyHex_1_NeuAc_1_, and HexNAc_3_Hex_3_DeoxyHex_1_NeuAc_3_. One potential reason is that the expression of this type of *O*-glycans is mainly driven by the level of fucosyltransferases [56], which is related to the downregulation of transcripts in Plasmax compared to DMEM. It should be noted that although FUT10 and FUT11 exhibited higher abundances among the fucosyltransferases, FUT10 has been considered as an enzyme that catalyzes the biosynthesis of Lewis X in biantennary *N*-glycans [57]. It has not been reported if they are involved in the biosynthesis of the Lewis X structure of *O*-glycans. In this study, focusing on the expression changes of glycans, we cannot draw a certain conclusion regarding whether these two enzymes are the main reasons for the abundance changes in sialyl-fucosylated glycans.

## 5. Summary

In this study, we investigated *N*- and *O*-glycosylation changes in cancer cell lines cultivated in a newly designed physiological medium Plasmax in relation to the two commercially available media DMEM and EMEM. For the breast cancer cell line MDA-MB-231BR, GlcNAc addition and the branching of *N*-glycans were more activated in Plasmax. However, the galactosylated *N*-glycans showed a higher relative abundance in the commercial media. The *O*-glycans of the 231BR cell line exhibited lower sialylation but higher sialo-fucosylation levels in Plasmax, indicating that Plasmax has different impacts on the enzymatic behaviors involved in *O*-glycosylation compared to *N*-glycans. On the other hand, the activation of the GlcNAc addition and branching of *N*-glycans was also observed in the brain cancer cell line CRL-1620, suggesting that these changes were more likely general effects on cancer cells cultivated in Plasmax. In addition, the sialylation levels and fucosylation levels of CRL *N*-glycans in Plasmax were significantly different than DMEM and EMEM, which resulted in higher expressions of sialo-fucosylated *N*-glycans in Plasmax than both commercial media. Compared to DMEM, both sialylated *O*-glycans and fucosylated *O*-glycans from the CRL cell line showed the same regulation trends as *N*-glycans. The glycan expressions changes were also correlated with the transcriptomics results, indicating that the unique environment of Plasmax can induce alterations in glycotransferases. For EMEM specifically, the lower number of unique *O*-glycan structures detected in this medium renders EMEM inadequate for cell line *O*-glycomic analysis. The glycomics analyses in this study provided complementary information to the original work that focused on colony formation, transcriptomics, and metabolomics. Since aberrant glycosylation has been related to a variety of diseases such as cancer, and significant glycan expression alterations were observed in this study, it is necessary to consider the influence of nutrient compositions of the culturing media and the selection of a suitable media for in vitro cancer cell glycomics.

## Figures and Tables

**Table 1:** Expression discrepancies of different types of *N*-glycans derived from 231BR and CRL cell lines in three media. The numbers in brackets are standard deviations (*n* = 3). *p*-values were calculated using Student’s *t*-tests to indicate the significant level of differences.

Glycan Type	Plasmax-DMEM	*p*-Value (Plasmax/DMEM)	Plasmax-EMEM	*p*-Value (Plasmax/EMEM)	EMEM-DMEM	*p*-Value (EMEM/DMEM)
**Expression Discrepancies of Different Types of *N*-Glycans Derived from MDA-MB-231BR Cell Line**						
High Mannose	+3.2% (± 3.3%)	0.36	−0.6% (±2.5%)	0.86	+3.8% (±2.1%)	0.02
Sialylated	+0.1% (±0.4%)	0.68	−0.6% (±0.6%)	0.38	+0.7% (±1.1%)	0.28
Fucosylated	−1.7% (±0.1%)	0.61	−0.5% (±2.6%)	0.90	−1.2% (±2.9%)	0.63
Sia & Fuc	−0.5% (±0.4%)	0.65	−2.9% (±2.6%)	0.26	+2.4% (±4.3%)	0.34
Other	−1.2% (±0.6%)	0.09	+4.6% (±0.4%)	2 × 10^−4^	−5.7% (±0.6%)	2 × 10^−4^
**Expression Discrepancies of Different Types of *N*-Glycans Derived from CRL-1620 Cell Line**						
High Mannose	−2.7% (±3.1%)	0.20	−5.0% (±0.7%)	3 × 10^−4^	+2.4% (±1.5%)	0.25
Sialylated	+1.9% (±0.3%)	1 × 10^−3^	−0.1% (±0.2%)	0.80	+2.0% (±0.3%)	1 × 10^−3^
Fucosylated	−4.3% (±2.3%)	0.04	+3.2% (±1.3%)	0.04	−7.5% (±3.6%)	0.01
Sia & Fuc	+2.5% (±0.6%)	9 × 10^−4^	+0.5% (±0.3%)	0.06	+1.9% (±0.3%)	2 × 10^−3^
Other	+2.8% (±0.4%)	1 × 10^−3^	+1.5% (±0.4%)	0.02	+1.3% (±0.2%)	0.06

**Table 2:** Expression discrepancies of different types of *O*-glycans derived from the 231BR and CRL cell lines in three media. The numbers in brackets are standard deviations (*n* = 3). *p*-values were calculated using Student’s *t*-tests to indicate the significant level of differences.

Glycan Type	Plasmax-DMEM	*p*-Value (Plasmax/DMEM)	Plasmax-EMEM	*p*-Value (Plasmax/EMEM)	EMEM-DMEM	*p*-Value (EMEM/DMEM)
**Expression Discrepancies of Different Types of *O*-Glycans Derived from MDA-MB-231BR Cell Line**						
Sialylated	−6.4% (±1.7%)	4 × 10^−3^	+3.4% (±0.9%)	0.05	−9.8% (±1.0%)	7 × 10^−4^
Fucosylated	−1.4% (±0.8%)	0.40	+8.1% (±1.0%)	1 × 10^−4^	−9.6% (±2.0%)	3 × 10^−3^
Sia & Fuc	+16.3% (±7.9%)	0.01	+2.8% (±8.7%)	0.74	+13.2% (±11.7%)	0.15
Other	−8.2% (±4.1%)	0.06	−14.3% (±13.8%)	0.16	+6.1% (±12.2%)	0.48
**Expression Discrepancies of Different Types of *O*-Glycans Derived from CRL-1620 Cell Line**						
Sialylated	+15.4% (±6.0%)	0.01	+6.7% (±6.0%)	0.24	+8.7% (±6.1%)	0.06
Fucosylated	−6.2% (±2.2%)	4 × 10^−3^	−6.3% (±0.7%)	5 × 10^−5^	+0.1% (±0.7%)	0.95
Sia & Fuc	−10.1% (±3.9%)	0.02	−27.9% (±1.6%)	1 × 10^−4^	+17.8% (±5.0%)	4 × 10^−3^
Other	+1.0% (±1.0%)	0.86	+27.5% (±5.3%)	3 × 10^−3^	−26.5% (±4.4%)	3 × 10^−3^

## Data Availability

The research data used in this article are available from the corresponding author upon request.

## Supplementary Materials

The following supporting information can be downloaded at: https://www.mdpi.com/article/10.3390/biom12060743/s1. Table S1: Full list of the formulation of Plasmax, Table S2: Major nutrients and metabolites with different amounts in Plasmax, EMEM and DMEM. The commercial media include high abundance of Glucose, Glutamine, etc., while Plasmax mimics the concentration of human blood, and includes metabolites such as Urea and Urate, Table S3: Relative abundance of glycans from 231BR cell line, Table S4: Relative abundance of glycans from CRL cell line, Table S5: RPKM of glycogenes expressed in 231BR and CRL cell lines cultivated by three media, Figure S1: Nutritional composition of three media: Plasmax, EMEM, and DMEM, Figure S2: Examples of positional structural identification method *N*-glycans, Figure S3: Heatmaps of significant *N*-glycans from 231BR cell line and CRL cell line, Figure S4: Heatmaps of significant *O*-glycans from 231BR cell line and CRL cell line, Figures S5–S7: RPKM of transcript expressions of glycosyltransferases that correlated with changes in glycomes, and glycan synthesis pathways.
