# Supplementary material for: Glycome Profiling of Cancer Cell Lines Cultivated in Physiological and Commercial Media
Source: Biomolecules. 2022 May 24;12(6):743. doi: 10.3390/biom12060743 (PMC9221004; doi:10.3390/biom12060743)
Supplement: Supplementary file 1 [file biomolecules-12-00743-s001.zip › biomolecules-1706791-supplementary.pdf]

# Supporting Information

## Glycome Profiling of Cancer Cell Lines Cultivated in Physiological and Commercial Media

Junyao Wang[1], Wenjing Peng[1], Aiying Yu[1], Mohamed Fokar[2] and Yehia Mechref[1,2*]

[1] Department of Chemistry and Biochemistry, Texas Tech University, Lubbock TX, 79409
[2] Center of Biotechnology and Genomics, Texas Tech University, Lubbock TX, 79409
Junyao.Wang@ttu.edu (J.W.); Wenjing.Peng@ttu.edu (W.P.); Aiying.Yu@ttu.edu (A.Y.); M.Fokar@ttu.edu (M.F.).
*Correspondence: Yehia.Mechref@ttu.edu (Y.M.); Tel: 806-742-3059

**Keywords:** Glycomics, Culture media, Cancer cell line, Differential expression analysis, LC-MS/MS

## Table of Contents

**Table S1.** Full list of the formulation of Plasmax.

**Table S2.** Major nutrients and metabolites with different amounts in Plasmax, EMEM and DMEM.

**Table S3.** Relative abundance of **(A)** *N*-glycans and **(B)** *O*-glycans from 231BR cell line.

**Table S4.** Relative abundance of **(A)** *N*-glycans and **(B)** *O*-glycans from CRL cell line.

**Figure S1.** Nutritional composition of three media: **(A)** Plasmax, **(B)** EMEM, and **(C)** DMEM.

**Figure S2.** Examples of positional structural identification method of **(A)** *N*-glycan $HexNAc_4Hex_3DeoxyHex_1$ from 231BR cell line and **(C)** *N*-glycan HexNAc4Hex5DeoxyHex1 from CRL cell line using LC-MS/MS. Insets of **(A)** and **(C)** are the full MS spectra, **(B)** and **(D)** are MS/MS spectra with identified fragment ions assigned to corresponding peaks. Symbols: ■, N-acetylglucosamine (GlcNAc); ○, Galactose (Gal); ▼, Fucose (Fuc); ●, Mannose (Man).

**Figure S3.** Heatmaps of significant *N*-glycans from **(A)** 231BR cell line and **(B)** CRL cell line. Red color denotes up-regulation, green color denotes down-regulation.

**Figure S4.** Heatmaps of significant *O*-glycans from **(A)** 231BR cell line and **(B)** CRL cell line. Color codes are the same as **Figure S3**.

**Figure S5.** Transcript expressions of 231BR cell line. **(A)** Galactosyltransferases in DMEM and Plasmax; **(B)** *N*-acetylglucosaminyltransferases in EMEM and Plasmax. **(C)** Synthesis pathways of complex capping *N*- and *O*-glycans. The red box highlights the processes of connecting galactose to GlcNAc, catalyzed by B4GALT/B3GALT. **(D)** Synthesis pathways of *N*-glycan processing and branching. Red boxes highlight the processes of attaching GlcNAc to mannose on core structure, catalyzed by MGATs. The synthesis pathways are acquired from GlycoMaple (https://glycosmos.org/glycomaple/index).

**Figure S6.** Transcript expressions of CRL cell line. **(A)** *N*-acetylglucosaminyltransferases in EMEM and Plasmax; **(B)** Fucosyltransferases in EMEM and Plasmax. **(C)** Synthesis pathways of *N*-glycan processing and branching. The labeled transcripts of transferases depict the processes of attaching GlcNAc to mannose on core structure, and core-fucosylation, catalyzed by MGATs, and FUT8, respectively. The synthesis pathways are acquired from GlycoMaple (https://glycosmos.org/glycomaple/index).

**Figure S7.** Transcript expressions of **(A)** Sialytransferases in 231BR cell line cultivated by DMEM and Plasmax; **(B)** Fucosyltransferases in CRL cell line cultivated by DMEM and Plasmax. **(C)** Synthesis pathways of complex capping *N*- and *O*-glycans. The labeled transcripts of transferases depict the processes of sialylation catalyzed by ST3GAL or ST6GAL, and fucosylation catalyzed by FUTs. The synthesis pathways are acquired from GlycoMaple (https://glycosmos.org/glycomaple/index).

**Table S1.** Full list of the formulation of Plasmax.

| No. | | (g/mol) | C (μM) | Weight for 1L(mg) |
|---|---|---|---|---|
| 1 | L-Alanine | 89.09 | 510 | 45.44 |
| | L-Arginine | 174.20 | 64 | 11.15 |
| | L-Asparagine | 132.12 | 41 | 5.42 |
| | L-Aspartic acid | 133.10 | 6 | 0.80 |
| | L-Glutamate | 187.13 | 98 | 18.34 |
| | Glycine | 75.07 | 330 | 24.77 |
| | L-Histidine | 155.15 | 120 | 18.62 |
| | L-Isoleucine | 131.17 | 140 | 18.36 |
| | L-Leucine | 131.17 | 170 | 22.30 |
| | L-Lysine | 146.19 | 220 | 32.16 |
| | L-Methionine | 149.21 | 30 | 4.48 |
| | L-Phenylalanine | 165.19 | 68 | 11.23 |
| | L-Proline | 115.13 | 360 | 41.45 |
| | L-Serine | 105.09 | 140 | 14.71 |
| | L-Threonine | 119.12 | 240 | 28.59 |
| | L-Tryptophan | 204.23 | 78 | 15.93 |
| | L-Tyrosine | 181.19 | 74 | 13.41 |
| | L-Valine | 117.15 | 230 | 26.94 |
| | L-Citrulline | 175.19 | 55 | 9.64 |
| | L-Cystine | 240.30 | 65 | 15.62 |
| | L-Ornithine | 168.62 | 80 | 13.49 |
| 2 | L-Cysteine | 121.16 | 33 | 4.00 |
| | (DL-2-Aminobutyric acid) | 103.12 | 41 | 4.23 |
| | L-Homocysteine | 135.18 | 9 | 1.22 |
| | 4-Hydroxy-L-proline | 131.13 | 13 | 1.70 |
| | L-Pyroglutamate | 129.11 | 20 | 2.58 |
| | L-Acetyl glycine | 117.10 | 70 | 8.20 |
| | L-Carnosine | 226.23 | 6 | 1.36 |
| | Glutathione (reduced) | 307.32 | 37 | 11.37 |
| | Taurine | 125.15 | 130 | 16.27 |
| | (Betaine) | 117.15 | 72 | 8.43 |
| | Acetate | 82.03 | 42 | 3.45 |
| | Acetone | 58.08 | 55 | 3.19 |
| | (O-Acetyl-L-carnitine hydrochloride) | 239.70 | 5 | 1.20 |
| | (sodium citrate dihydrate) | 294.10 | 114 | 33.53 |
| | Carnitine | 197.66 | 46 | 9.09 |

| No. | | (g/mol) | C (µM) | Weight for 1L(mg) |
|---|---|---|---|---|
| 2 | Creatine | 131.13 | 37 | 4.85 |
| | Creatinine | 113.12 | 74 | 8.37 |
| | (sodium formate) | 68.01 | 33 | 2.24 |
| | Glycerol | 92.09 | 82 | 7.55 |
| | (2-hydroxybutyric acid sodium salt) | 126.09 | 31 | 3.91 |
| | ((+-)-sodium 3-hydroxybutyrate) | 126.09 | 77 | 9.71 |
| | ((+-)-sodium B-hydroxyisobutyrate) | 126.09 | 20 | 2.52 |
| | Hypoxanthine | 136.11 | 5 | 0.68 |
| | Lactate | 112.06 | 500 | 56.03 |
| | Methyl acetoacetate | 116.12 | 41 | 4.76 |
| | (di-sodium succinate) | 162.05 | 23 | 3.73 |
| | Uracil | 112.09 | 2 | 0.22 |
| | Urea | 60.06 | 3000 | 180.17 |
| | Uridine | 244.20 | 3 | 0.73 |
| 3 | Ammonium Chloride | 53.49 | 50 | 26.74 |
| | Ferric Sulfate | 399.88 | 1.0428 | 4.17 |
| | Zinc Sulfate | 287.56 | 1.5 | 4.31 |
| | Ammonium Metavanadate | 116.98 | 0.0026 | 3.04 |
| | (copper(II) sulfate) | 159.61 | 0.0052 | 8.30 |
| | Ferric Nitrate | 404.00 | 0.1238 | 500.15 |
| | Manganous Chloride | 125.84 | 0.0002 | 0.25 |
| | Sodium Selenite | 172.94 | 0.0289 | 49.98 |
| 4 | Urate | 190.09 | 270 | 256.62 |
| 5 | L-Glutamine | 146.15 | 650 | 94.99 |
| | BME vitamins | - | - | - |
| 6 | Ascorbate | 198.11 | 62 | 1228.28 |
| | Vitamin B12 | 1355.37 | 0.005 | 0.68 |
| 7 | EBSS | - | - | - |
| 8 | (sodium pyruvate) | 110.04 | 100.00 | 11.00 |

**Table S2.** Major nutrients and metabolites with different amounts in Plasmax, EMEM and DMEM. The commercial media include high abundance of Glucose, Glutamine, *etc.*, while Plasmax mimics the concentration of human blood, and includes metabolites such as Urea and Urate.

| Nutrient | Plasmax | EMEM | DMEM |
|---|---|---|---|
| D-Glucose | 38.5% | 43.5% | 67.8% |
| Urea | 20.6% | - | - |
| L-Glutamine | 4.5% | 15.7% | 10.8% |
| Lactate | 3.5% | - | - |
| Urate | 1.9% | - | - |
| L-Threonine | 1.7% | 3.1% | 2.2% |
| L-Lysine | 1.5% | 3.1% | 2.2% |
| L-Valine | 1.6% | 3.1% | 2.2% |
| Glycine | 2.3% | 0.8% | 1.1% |
| Sodium Pyruvate | 0.7% | 7.8% | 2.7% |

**Table S3A.** Relative abundance of *N*-glycans from 231BR cell line.

| N-glycan | Theoretical m/z | Observed m/z | Mass Accuracy (ppm) | DMEM_1 | DMEM_2 | DMEM_3 | Avg | StdV | EMEM_1 | EMEM_2 | EMEM_3 | Avg | StdV | Plasmax_1 | Plasmax_2 | Plasmax_3 | Avg | StdV | P value DMEM EMEM | P value DMEM Plasmax | P value EMEM Plasmax |
|---|---|---|---|---|---|---|---|---|---|---|---|---|---|---|---|---|---|---|---|---|---|
| 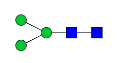 | 1165.6331 | 1165.6321 | 0.86 | 0.14534 | 0.11589 | 0.12332 | 0.12819 | 0.01531 | 0.13142 | 0.15393 | 0.14448 | 0.14328 | 0.01130 | 0.13446 | 0.11741 | 0.12798 | 0.12662 | 0.00861 | 2.E-01 | 9.E-01 | 1.E-01 |
| 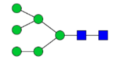 | 889.4702 | 889.4701 | 0.11 | 0.04093 | 0.06929 | 0.06166 | 0.05729 | 0.01467 | 0.05252 | 0.06470 | 0.06486 | 0.06069 | 0.00708 | 0.08719 | 0.04621 | 0.10012 | 0.07784 | 0.02815 | 7.E-01 | 3.E-01 | 4.E-01 |
| 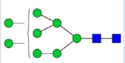 | 1093.5700 | 1093.5714 | 1.28 | 0.01909 | 0.02418 | 0.02410 | 0.02246 | 0.00291 | 0.03726 | 0.02527 | 0.02982 | 0.03078 | 0.00605 | 0.03606 | 0.02758 | 0.02703 | 0.03023 | 0.00506 | 1.E-01 | 8.E-02 | 9.E-01 |
| 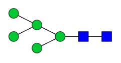 | 787.4203 | 787.4194 | 1.14 | 0.02949 | 0.04276 | 0.04173 | 0.03799 | 0.00738 | 0.04075 | 0.03681 | 0.03340 | 0.03698 | 0.00368 | 0.03390 | 0.02595 | 0.03613 | 0.03199 | 0.00535 | 8.E-01 | 3.E-01 | 3.E-01 |
| 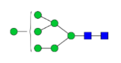 | 991.5201 | 991.5196 | 0.50 | 0.01804 | 0.02805 | 0.03018 | 0.02542 | 0.00648 | 0.03918 | 0.02479 | 0.02135 | 0.02844 | 0.00946 | 0.04125 | 0.02488 | 0.02436 | 0.03016 | 0.00961 | 7.E-01 | 5.E-01 | 8.E-01 |
| 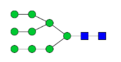 | 1195.6199 | 1195.6218 | 1.59 | 0.01195 | 0.01051 | 0.01193 | 0.01147 | 0.00082 | 0.02660 | 0.01803 | 0.02147 | 0.02204 | 0.00431 | 0.02764 | 0.01956 | 0.01676 | 0.02132 | 0.00565 | 1.E-02 | 4.E-02 | 9.E-01 |
| 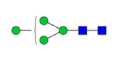 | 1369.7329 | 1369.7328 | 0.07 | 0.01020 | 0.00978 | 0.00943 | 0.00981 | 0.00039 | 0.00822 | 0.00821 | 0.00771 | 0.00805 | 0.00029 | 0.00648 | 0.00560 | 0.00683 | 0.00630 | 0.00063 | 3.E-03 | 1.E-03 | 1.E-02 |
| 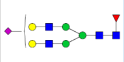 | 867.1213 | 867.1216 | 0.31 | 0.02426 | 0.02651 | 0.01343 | 0.02140 | 0.00699 | 0.01991 | 0.02534 | 0.04915 | 0.03147 | 0.01556 | 0.01403 | 0.01773 | 0.02319 | 0.01832 | 0.00461 | 4.E-01 | 6.E-01 | 2.E-01 |
| 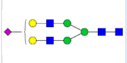 | 809.0916 | 809.0909 | 0.87 | 0.00826 | 0.00812 | 0.00441 | 0.00693 | 0.00219 | 0.00698 | 0.00877 | 0.01721 | 0.01099 | 0.00546 | 0.00508 | 0.00708 | 0.01064 | 0.00760 | 0.00282 | 3.E-01 | 8.E-01 | 4.E-01 |
| 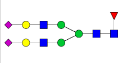 | 987.5126 | 987.5132 | 0.64 | 0.00963 | 0.00865 | 0.00257 | 0.00695 | 0.00382 | 0.00532 | 0.01037 | 0.03027 | 0.01532 | 0.01319 | 0.00352 | 0.00504 | 0.01033 | 0.00630 | 0.00357 | 4.E-01 | 8.E-01 | 3.E-01 |
| 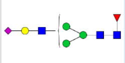 | 1075.5650 | 1075.5642 | 0.74 | 0.00475 | 0.00459 | 0.00203 | 0.00379 | 0.00153 | 0.00324 | 0.00548 | 0.01103 | 0.00659 | 0.00401 | 0.00214 | 0.00314 | 0.00508 | 0.00345 | 0.00149 | 3.E-01 | 8.E-01 | 3.E-01 |
| 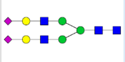 | 929.4828 | 929.4824 | 0.47 | 0.00147 | 0.00153 | 0.00046 | 0.00115 | 0.00060 | 0.00096 | 0.00234 | 0.00733 | 0.00354 | 0.00335 | 0.00085 | 0.00101 | 0.00261 | 0.00149 | 0.00097 | 3.E-01 | 6.E-01 | 4.E-01 |
| 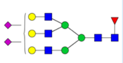 | 1137.2546 | 1137.2554 | 0.70 | 0.00115 | 0.00104 | 0.00029 | 0.00083 | 0.00046 | 0.00097 | 0.00176 | 0.00504 | 0.00259 | 0.00216 | 0.00042 | 0.00055 | 0.00104 | 0.00067 | 0.00033 | 2.E-01 | 7.E-01 | 2.E-01 |
| 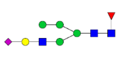 | 1177.6149 | 1177.6148 | 0.08 | 0.00054 | 0.00063 | 0.00034 | 0.00051 | 0.00015 | 0.00050 | 0.00060 | 0.00121 | 0.00077 | 0.00039 | 0.00000 | 0.00012 | 0.00040 | 0.00018 | 0.00021 | 3.E-01 | 9.E-02 | 8.E-02 |
| 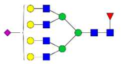 | 1166.6054 | 1166.6059 | 0.43 | 0.00036 | 0.00027 | 0.00012 | 0.00025 | 0.00012 | 0.00051 | 0.00037 | 0.00086 | 0.00058 | 0.00025 | 0.00028 | 0.00038 | 0.00028 | 0.00031 | 0.00006 | 1.E-01 | 4.E-01 | 1.E-01 |
| 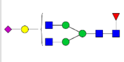 | 1198.1282 | 1198.1278 | 0.29 | 0.00040 | 0.00048 | 0.00030 | 0.00039 | 0.00009 | 0.00021 | 0.00036 | 0.00080 | 0.00046 | 0.00031 | 0.00015 | 0.00018 | 0.00052 | 0.00028 | 0.00021 | 7.E-01 | 5.E-01 | 5.E-01 |
| 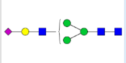 | 988.5204 | 988.5196 | 0.81 | 0.00021 | 0.00040 | 0.00011 | 0.00024 | 0.00015 | 0.00019 | 0.00023 | 0.00113 | 0.00052 | 0.00053 | 0.00011 | 0.00017 | 0.00025 | 0.00018 | 0.00007 | 4.E-01 | 5.E-01 | 3.E-01 |
| 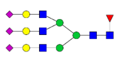 | 1257.6458 | 1257.6468 | 0.77 | 0.00006 | 0.00004 | 0.00001 | 0.00004 | 0.00002 | 0.00004 | 0.00011 | 0.00063 | 0.00026 | 0.00032 | 0.00001 | 0.00001 | 0.00007 | 0.00003 | 0.00003 | 3.E-01 | 8.E-01 | 3.E-01 |
| 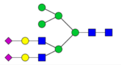 | 1065.5494 | 1065.5432 | 5.79 | 0.00000 | 0.00005 | 0.00010 | 0.00005 | 0.00005 | 0.00007 | 0.00037 | 0.00000 | 0.00015 | 0.00019 | 0.00010 | 0.00087 | 0.00000 | 0.00032 | 0.00048 | 5.E-01 | 4.E-01 | 6.E-01 |

| N-glycan | Theoretical m/z | Observed m/z | Mass Accuracy (ppm) | DMEM_1 | DMEM_2 | DMEM_3 | Avg | StdV | EMEM_1 | EMEM_2 | EMEM_3 | Avg | StdV | Plasmax_1 | Plasmax_2 | Plasmax_3 | Avg | StdV | P value DMEM EMEM | P value DMEM Plasmax | P value EMEM Plasmax |
|---|---|---|---|---|---|---|---|---|---|---|---|---|---|---|---|---|---|---|---|---|---|
| 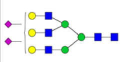 | 1079.2249 | 1079.2255 | 0.59 | 0.00002 | 0.00003 | 0.00001 | 0.00002 | 0.00001 | 0.00004 | 0.00008 | 0.00022 | 0.00012 | 0.00009 | 0.00002 | 0.00000 | 0.00006 | 0.00003 | 0.00003 | 2.E-01 | 8.E-01 | 2.E-01 |
| 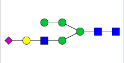 | 1090.5703 | 1090.5707 | 0.37 | 0.00016 | 0.00021 | 0.00007 | 0.00015 | 0.00007 | 0.00014 | 0.00026 | 0.00048 | 0.00029 | 0.00017 | 0.00000 | 0.00015 | 0.00022 | 0.00012 | 0.00011 | 3.E-01 | 8.E-01 | 2.E-01 |
| 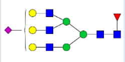 | 1016.8634 | 1016.8635 | 0.13 | 0.00046 | 0.00044 | 0.00031 | 0.00040 | 0.00008 | 0.00049 | 0.00056 | 0.00121 | 0.00075 | 0.00040 | 0.00019 | 0.00036 | 0.00028 | 0.00028 | 0.00009 | 2.E-01 | 1.E-01 | 1.E-01 |
| 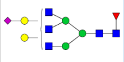 | 948.8301 | 948.8303 | 0.21 | 0.00001 | 0.00003 | 0.00000 | 0.00001 | 0.00001 | 0.00000 | 0.00000 | 0.00000 | 0.00000 | 0.00000 | 0.00000 | 0.00002 | 0.00004 | 0.00002 | 0.00002 | 2.E-01 | 6.E-01 | 1.E-01 |
| 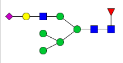 | 853.4458 | 853.4468 | 1.13 | 0.00000 | 0.00000 | 0.00000 | 0.00000 | 0.00000 | 0.00015 | 0.00000 | 0.00006 | 0.00007 | 0.00007 | 0.00000 | 0.00001 | 0.00004 | 0.00002 | 0.00002 | 2.E-01 | 2.E-01 | 3.E-01 |
| 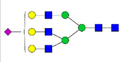 | 958.8336 | 958.8334 | 0.24 | 0.00000 | 0.00000 | 0.00000 | 0.00000 | 0.00000 | 0.00003 | 0.00005 | 0.00009 | 0.00006 | 0.00003 | 0.00003 | 0.00004 | 0.00005 | 0.00004 | 0.00001 | 3.E-02 | 7.E-04 | 4.E-01 |
| 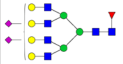 | 1286.9966 | 1286.9981 | 1.14 | 0.00000 | 0.00000 | 0.00000 | 0.00000 | 0.00000 | 0.00002 | 0.00002 | 0.00006 | 0.00004 | 0.00002 | 0.00001 | 0.00000 | 0.00002 | 0.00001 | 0.00001 | 7.E-02 | 2.E-01 | 1.E-01 |
| 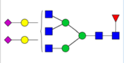 | 1069.2213 | 1069.2228 | 1.37 | 0.00002 | 0.00001 | 0.00000 | 0.00001 | 0.00001 | 0.00000 | 0.00000 | 0.00000 | 0.00000 | 0.00000 | 0.00000 | 0.00000 | 0.00000 | 0.00000 | 0.00000 | 2.E-01 | 2.E-01 | - |
| 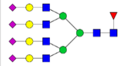 | 1146.0863 | 1146.0882 | 1.66 | 0.00002 | 0.00001 | 0.00000 | 0.00001 | 0.00001 | 0.00000 | 0.00000 | 0.00000 | 0.00000 | 0.00000 | 0.00000 | 0.00000 | 0.00000 | 0.00000 | 0.00000 | 2.E-01 | 2.E-01 | - |
| 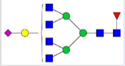 | 962.5056 | 962.5058 | 0.21 | 0.00002 | 0.00000 | 0.00001 | 0.00001 | 0.00001 | 0.00000 | 0.00000 | 0.00000 | 0.00000 | 0.00000 | 0.00000 | 0.00000 | 0.00000 | 0.00000 | 0.00000 | 2.E-01 | 2.E-01 | - |
| 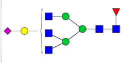 | 1320.6913 | 1320.6900 | 0.98 | 0.00006 | 0.00004 | 0.00000 | 0.00003 | 0.00003 | 0.00000 | 0.00000 | 0.00000 | 0.00000 | 0.00000 | 0.00000 | 0.00000 | 0.00000 | 0.00000 | 0.00000 | 1.E-01 | 1.E-01 | - |
| 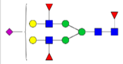 | 983.1808 | 983.1754 | 5.49 | 0.00000 | 0.00000 | 0.00000 | 0.00000 | 0.00000 | 0.00000 | 0.00000 | 0.00000 | 0.00000 | 0.00000 | 0.00001 | 0.00002 | 0.00000 | 0.00001 | 0.00001 | - | 1.E-01 | 1.E-01 |
| 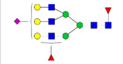 | 1074.8931 | 1074.8876 | 5.12 | 0.00000 | 0.00000 | 0.00000 | 0.00000 | 0.00000 | 0.00000 | 0.00000 | 0.00000 | 0.00000 | 0.00000 | 0.00000 | 0.00002 | 0.00001 | 0.00001 | 0.00001 | - | 1.E-01 | 1.E-01 |
| 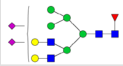 | 1123.5791 | 1123.5732 | 5.25 | 0.00000 | 0.00000 | 0.00000 | 0.00000 | 0.00000 | 0.00002 | 0.00001 | 0.00000 | 0.00001 | 0.00001 | 0.00000 | 0.00000 | 0.00000 | 0.00000 | 0.00000 | 1.E-01 | - | 1.E-01 |
| 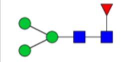 | 1339.7223 | 1339.7212 | 0.82 | 0.29531 | 0.28459 | 0.32425 | 0.30139 | 0.02051 | 0.38400 | 0.37973 | 0.33742 | 0.36705 | 0.02575 | 0.22729 | 0.24679 | 0.23455 | 0.23621 | 0.00986 | 3.E-02 | 8.E-03 | 1.E-03 |
| 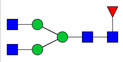 | 915.4914 | 915.4902 | 1.31 | 0.11912 | 0.11270 | 0.08856 | 0.10679 | 0.01611 | 0.09842 | 0.09476 | 0.08015 | 0.09111 | 0.00967 | 0.14314 | 0.17611 | 0.13621 | 0.15182 | 0.02132 | 2.E-01 | 4.E-02 | 1.E-02 |
| 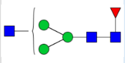 | 792.9283 | 792.9270 | 1.58 | 0.07048 | 0.07274 | 0.07440 | 0.07254 | 0.00197 | 0.03118 | 0.04154 | 0.04207 | 0.03827 | 0.00614 | 0.06719 | 0.08499 | 0.06948 | 0.07389 | 0.00968 | 8.E-04 | 8.E-01 | 6.E-03 |
| 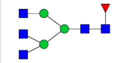 | 1038.0546 | 1038.0542 | 0.34 | 0.02775 | 0.03145 | 0.03265 | 0.03062 | 0.00255 | 0.03108 | 0.02551 | 0.02251 | 0.02637 | 0.00435 | 0.03888 | 0.04873 | 0.04442 | 0.04401 | 0.00494 | 2.E-01 | 1.E-02 | 1.E-02 |
| 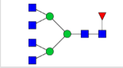 | 1160.6177 | 1160.6165 | 1.03 | 0.01661 | 0.01621 | 0.01846 | 0.01709 | 0.00120 | 0.01147 | 0.00976 | 0.00777 | 0.00967 | 0.00185 | 0.02605 | 0.03423 | 0.02885 | 0.02971 | 0.00416 | 4.E-03 | 7.E-03 | 2.E-03 |
| 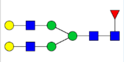 | 1119.5912 | 1119.5897 | 1.34 | 0.02423 | 0.02438 | 0.02424 | 0.02428 | 0.00009 | 0.02026 | 0.01492 | 0.01659 | 0.01726 | 0.00273 | 0.01133 | 0.01337 | 0.01181 | 0.01217 | 0.00107 | 1.E-02 | 4.E-05 | 4.E-02 |
| 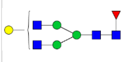 | 1017.5413 | 1017.5414 | 0.10 | 0.01340 | 0.01310 | 0.01411 | 0.01354 | 0.00052 | 0.00821 | 0.00744 | 0.00723 | 0.00763 | 0.00052 | 0.00682 | 0.00849 | 0.00746 | 0.00759 | 0.00084 | 2.E-04 | 5.E-04 | 1.E+00 |

| N-glycan | Theoretical m/z | Observed m/z | Mass Accuracy (ppm) | DMEM_1 | DMEM_2 | DMEM_3 | Avg | StdV | EMEM_1 | EMEM_2 | EMEM_3 | Avg | StdV | Plasmax_1 | Plasmax_2 | Plasmax_3 | Avg | StdV | P value DMEM EMEM | P value DMEM Plasmax | P value EMEM Plasmax |
|---|---|---|---|---|---|---|---|---|---|---|---|---|---|---|---|---|---|---|---|---|---|
| 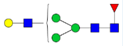 | 894.9782 | 894.9777 | 0.50 | 0.00914 | 0.00845 | 0.00974 | 0.00911 | 0.00065 | 0.00572 | 0.00809 | 0.00848 | 0.00743 | 0.00149 | 0.00408 | 0.00509 | 0.00377 | 0.00432 | 0.00069 | 1.E-01 | 9.E-04 | 3.E-02 |
| 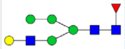 | 997.0281 | 997.0266 | 1.45 | 0.00300 | 0.00289 | 0.00325 | 0.00305 | 0.00018 | 0.00200 | 0.00185 | 0.00179 | 0.00188 | 0.00011 | 0.00169 | 0.00222 | 0.00193 | 0.00195 | 0.00026 | 7.E-04 | 4.E-03 | 7.E-01 |
| 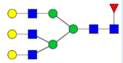 | 896.4721 | 896.4724 | 0.30 | 0.00026 | 0.00034 | 0.00000 | 0.00020 | 0.00018 | 0.00033 | 0.00011 | 0.00017 | 0.00020 | 0.00011 | 0.00020 | 0.00031 | 0.00012 | 0.00021 | 0.00009 | 1.E+00 | 9.E-01 | 9.E-01 |
| 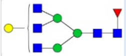 | 1140.1045 | 1140.1035 | 0.83 | 0.00022 | 0.00023 | 0.00025 | 0.00023 | 0.00001 | 0.00022 | 0.00026 | 0.00017 | 0.00022 | 0.00004 | 0.00008 | 0.00024 | 0.00017 | 0.00016 | 0.00008 | 5.E-01 | 2.E-01 | 3.E-01 |
| 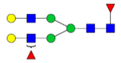 | 1206.6358 | 1206.6353 | 0.41 | 0.00007 | 0.00000 | 0.00004 | 0.00004 | 0.00004 | 0.00009 | 0.00004 | 0.00007 | 0.00007 | 0.00002 | 0.00014 | 0.00006 | 0.00006 | 0.00009 | 0.00004 | 3.E-01 | 2.E-01 | 6.E-01 |
| 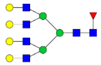 | 1046.2142 | 1046.2155 | 1.27 | 0.00070 | 0.00051 | 0.00051 | 0.00057 | 0.00011 | 0.00118 | 0.00044 | 0.00039 | 0.00067 | 0.00044 | 0.00070 | 0.00087 | 0.00024 | 0.00061 | 0.00033 | 7.E-01 | 9.E-01 | 9.E-01 |
| 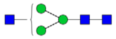 | 705.8837 | 705.8820 | 2.34 | 0.06007 | 0.05100 | 0.05307 | 0.05471 | 0.00476 | 0.01052 | 0.01000 | 0.00873 | 0.00975 | 0.00092 | 0.04991 | 0.04665 | 0.04149 | 0.04602 | 0.00425 | 9.E-05 | 8.E-02 | 1.E-04 |
| 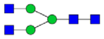 | 828.4468 | 828.4458 | 1.17 | 0.02438 | 0.02011 | 0.02135 | 0.02195 | 0.00220 | 0.01105 | 0.00946 | 0.00988 | 0.01013 | 0.00083 | 0.02145 | 0.01998 | 0.01762 | 0.01968 | 0.00193 | 1.E-03 | 3.E-01 | 1.E-03 |
| 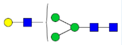 | 807.9336 | 807.9330 | 0.68 | 0.00269 | 0.00253 | 0.00288 | 0.00270 | 0.00018 | 0.00318 | 0.00344 | 0.00340 | 0.00334 | 0.00014 | 0.00256 | 0.00256 | 0.00260 | 0.00257 | 0.00003 | 8.E-03 | 3.E-01 | 8.E-04 |
| 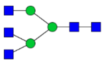 | 951.0100 | 951.0090 | 1.00 | 0.00244 | 0.00225 | 0.00210 | 0.00226 | 0.00017 | 0.00237 | 0.00171 | 0.00166 | 0.00191 | 0.00040 | 0.00189 | 0.00276 | 0.00239 | 0.00235 | 0.00044 | 2.E-01 | 8.E-01 | 3.E-01 |
| 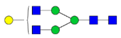 | 930.4967 | 930.4959 | 0.86 | 0.00272 | 0.00263 | 0.00289 | 0.00275 | 0.00013 | 0.00198 | 0.00181 | 0.00158 | 0.00179 | 0.00020 | 0.00173 | 0.00193 | 0.00200 | 0.00188 | 0.00014 | 2.E-03 | 2.E-03 | 5.E-01 |
| 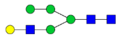 | 909.9835 | 909.9858 | 2.58 | 0.00000 | 0.00000 | 0.00000 | 0.00000 | 0.00000 | 0.00004 | 0.00000 | 0.00005 | 0.00003 | 0.00003 | 0.00000 | 0.00000 | 0.00000 | 0.00000 | 0.00000 | 1.E-01 | - | 1.E-01 |

**Table S3B.** Relative abundance of *O*-glycans from 231BR cell line.

| HexNac-Hex-deHex-NeuAc | Theoretical m/z | Observed m/z | Mass Accuracy (ppm) | DMEM_1 | DMEM_2 | DMEM_3 | Avg | StdV | EMEM_1 | EMEM_2 | EMEM_3 | Avg | StdV | Plasmax_1 | Plasmax_2 | Plasmax_3 | Avg | StdV | P value DMEM EMEM | P value DMEM Plasmax | P value EMEM Plasmax |
|---|---|---|---|---|---|---|---|---|---|---|---|---|---|---|---|---|---|---|---|---|---|
| **1-1-0-0** | 496.2759 | 496.2747 | 2.42 | 0.14092 | 0.12548 | 0.13383 | 0.13341 | 0.00773 | 0.20084 | 0.07218 | 0.14972 | 0.14091 | 0.06478 | 0.18388 | 0.10796 | 0.11889 | 0.13691 | 0.04105 | 9.E-01 | 9.E-01 | 9.E-01 |
| **1-1-0-1** | 857.4496 | 857.4486 | 1.17 | 0.05241 | 0.08641 | 0.07308 | 0.07063 | 0.01713 | 0.00000 | 0.00000 | 0.00000 | 0.00000 | 0.00000 | 0.04649 | 0.04676 | 0.04153 | 0.04493 | 0.00294 | 2.E-03 | 6.E-02 | 1.E-05 |
| **1-1-0-2** | 1218.6233 | 1218.6225 | 0.66 | 0.04644 | 0.05116 | 0.06372 | 0.05377 | 0.00893 | 0.00000 | 0.00000 | 0.00000 | 0.00000 | 0.00000 | 0.00000 | 0.00000 | 0.00000 | 0.00000 | 0.00000 | 5.E-04 | 5.E-04 | - |
| **1-1-1-0** | 670.3651 | 670.3642 | 1.34 | 0.11051 | 0.14842 | 0.16353 | 0.14082 | 0.02731 | 0.03552 | 0.02231 | 0.07429 | 0.04404 | 0.02702 | 0.10567 | 0.07384 | 0.11256 | 0.09736 | 0.02065 | 1.E-02 | 9.E-02 | 5.E-02 |
| **2-0-0-1** | 898.4761 | 898.4751 | 1.11 | 0.00000 | 0.00000 | 0.00000 | 0.00000 | 0.00000 | 0.06087 | 0.02981 | 0.01732 | 0.03600 | 0.02243 | 0.00724 | 0.00347 | 0.00593 | 0.00555 | 0.00191 | 5.E-02 | 7.E-03 | 8.E-02 |
| **2-0-1-1** | 1072.5653 | 1072.5597 | 5.22 | 0.00000 | 0.00000 | 0.00000 | 0.00000 | 0.00000 | 0.00000 | 0.00000 | 0.00000 | 0.00000 | 0.00000 | 0.07636 | 0.10316 | 0.14077 | 0.10676 | 0.03236 | - | 5.E-03 | 5.E-03 |
| **2-2-0-1** | 1306.6757 | 1306.6798 | 3.14 | 0.00499 | 0.00332 | 0.00314 | 0.00382 | 0.00102 | 0.00000 | 0.00000 | 0.00000 | 0.00000 | 0.00000 | 0.00441 | 0.00576 | 0.00401 | 0.00473 | 0.00092 | 3.E-03 | 3.E-01 | 9.E-04 |
| **2-2-0-2** | 834.4287 | 834.4326 | 4.73 | 0.08357 | 0.06962 | 0.05435 | 0.06918 | 0.01461 | 0.04803 | 0.09475 | 0.07836 | 0.07372 | 0.02370 | 0.08464 | 0.07477 | 0.06285 | 0.07409 | 0.01091 | 8.E-01 | 7.E-01 | 1.E+00 |
| **2-3-0-0** | 1149.6018 | 1149.6000 | 1.57 | 0.00000 | 0.00000 | 0.00000 | 0.00000 | 0.00000 | 0.04209 | 0.04229 | 0.15751 | 0.08063 | 0.06658 | 0.00000 | 0.00000 | 0.00000 | 0.00000 | 0.00000 | 1.E-01 | - | 1.E-01 |
| **2-3-1-0** | 1323.6910 | 1323.6919 | 0.68 | 0.00000 | 0.00000 | 0.00000 | 0.00000 | 0.00000 | 0.01602 | 0.01191 | 0.00723 | 0.01172 | 0.00440 | 0.00000 | 0.00000 | 0.00000 | 0.00000 | 0.00000 | 1.E-02 | - | 1.E-02 |
| **2-5-0-3** | 1321.1652 | 1321.1639 | 0.98 | 0.00430 | 0.00256 | 0.00405 | 0.00364 | 0.00094 | 0.00000 | 0.00000 | 0.00000 | 0.00000 | 0.00000 | 0.00299 | 0.00453 | 0.00403 | 0.00385 | 0.00078 | 3.E-03 | 8.E-01 | 1.E-03 |
| **2-6-0-0** | 881.4546 | 881.4527 | 2.10 | 0.01366 | 0.02731 | 0.03049 | 0.02382 | 0.00894 | 0.00000 | 0.00000 | 0.00000 | 0.00000 | 0.00000 | 0.00000 | 0.00000 | 0.00000 | 0.00000 | 0.00000 | 1.E-02 | 1.E-02 | - |
| **2-7-0-0** | 983.5045 | 983.5031 | 1.37 | 0.02693 | 0.03611 | 0.01996 | 0.02767 | 0.00810 | 0.01045 | 0.02830 | 0.02928 | 0.02268 | 0.01060 | 0.00000 | 0.00000 | 0.00000 | 0.00000 | 0.00000 | 6.E-01 | 4.E-03 | 2.E-02 |
| **2-8-0-0** | 1085.5544 | 1085.5535 | 0.78 | 0.06827 | 0.05631 | 0.03627 | 0.05362 | 0.01617 | 0.03719 | 0.06182 | 0.09174 | 0.06359 | 0.02732 | 0.03950 | 0.02322 | 0.03930 | 0.03401 | 0.00934 | 6.E-01 | 1.E-01 | 2.E-01 |
| **2-9-0-0** | 1187.6043 | 1187.6030 | 1.05 | 0.04799 | 0.03670 | 0.03780 | 0.04083 | 0.00622 | 0.02435 | 0.04250 | 0.07544 | 0.04743 | 0.02590 | 0.02591 | 0.02480 | 0.03884 | 0.02985 | 0.00781 | 7.E-01 | 1.E-01 | 3.E-01 |
| **2-10-0-0** | 1289.6542 | 1289.6527 | 1.12 | 0.00718 | 0.00602 | 0.00700 | 0.00673 | 0.00062 | 0.00000 | 0.00000 | 0.00000 | 0.00000 | 0.00000 | 0.00130 | 0.00112 | 0.00068 | 0.00104 | 0.00032 | 5.E-05 | 1.E-04 | 5.E-03 |
| **3-1-0-0** | 986.5285 | 986.5230 | 5.58 | 0.01027 | 0.00872 | 0.01107 | 0.01002 | 0.00120 | 0.00000 | 0.00000 | 0.00000 | 0.00000 | 0.00000 | 0.01181 | 0.00567 | 0.00214 | 0.00654 | 0.00489 | 1.E-04 | 3.E-01 | 8.E-02 |
| **3-2-1-0** | 1364.7175 | 1364.7095 | 5.86 | 0.03933 | 0.01608 | 0.02063 | 0.02535 | 0.01232 | 0.00000 | 0.00000 | 0.00000 | 0.00000 | 0.00000 | 0.06074 | 0.02659 | 0.03823 | 0.04185 | 0.01736 | 2.E-02 | 3.E-01 | 1.E-02 |
| **3-3-2-2** | 1233.1309 | 1233.1272 | 3.00 | 0.27838 | 0.22305 | 0.26468 | 0.25537 | 0.02882 | 0.44900 | 0.48766 | 0.24433 | 0.39366 | 0.13076 | 0.28405 | 0.37090 | 0.30358 | 0.31951 | 0.04557 | 1.E-01 | 1.E-01 | 4.E-01 |
| **3-4-1-1** | 1067.5494 | 1067.5485 | 0.80 | 0.00739 | 0.02083 | 0.01060 | 0.01294 | 0.00702 | 0.00260 | 0.00473 | 0.01322 | 0.00685 | 0.00562 | 0.00191 | 0.00200 | 0.00296 | 0.00229 | 0.00058 | 3.E-01 | 6.E-02 | 2.E-01 |
| **5-5-0-0** | 1147.0941 | 1147.0884 | 4.97 | 0.00654 | 0.00301 | 0.00250 | 0.00402 | 0.00220 | 0.00118 | 0.00307 | 0.00536 | 0.00320 | 0.00209 | 0.00218 | 0.00181 | 0.00361 | 0.00253 | 0.00095 | 7.E-01 | 3.E-01 | 6.E-01 |
| **5-6-0-0** | 1249.1440 | 1249.1406 | 2.72 | 0.00000 | 0.00000 | 0.00000 | 0.00000 | 0.00000 | 0.00176 | 0.00242 | 0.00352 | 0.00257 | 0.00089 | 0.00604 | 0.00465 | 0.00773 | 0.00614 | 0.00154 | 7.E-03 | 2.E-03 | 3.E-02 |
| **5-6-2-0** | 949.1581 | 949.1601 | 2.11 | 0.00000 | 0.00000 | 0.00000 | 0.00000 | 0.00000 | 0.00000 | 0.00000 | 0.00000 | 0.00000 | 0.00000 | 0.00146 | 0.00184 | 0.00150 | 0.00160 | 0.00021 | - | 2.E-04 | 2.E-04 |
| **6-5-0-0** | 1269.6573 | 1269.6597 | 1.93 | 0.00000 | 0.00000 | 0.00000 | 0.00000 | 0.00000 | 0.00000 | 0.00000 | 0.00000 | 0.00000 | 0.00000 | 0.00007 | 0.00159 | 0.00044 | 0.00070 | 0.00079 | - | 2.E-01 | 2.E-01 |
| **6-5-0-1** | 967.1654 | 967.1700 | 4.79 | 0.00615 | 0.00646 | 0.00629 | 0.00630 | 0.00016 | 0.00000 | 0.00000 | 0.00000 | 0.00000 | 0.00000 | 0.00791 | 0.01602 | 0.00822 | 0.01072 | 0.00459 | 3.E-07 | 2.E-01 | 2.E-02 |
| **6-5-1-0** | 904.8039 | 904.8074 | 3.91 | 0.03803 | 0.04609 | 0.04800 | 0.04404 | 0.00529 | 0.05844 | 0.08655 | 0.03513 | 0.06004 | 0.02575 | 0.03762 | 0.08329 | 0.05405 | 0.05832 | 0.02313 | 4.E-01 | 4.E-01 | 9.E-01 |
| **6-6-1-0** | 972.8371 | 972.8339 | 3.32 | 0.00671 | 0.02633 | 0.00899 | 0.01401 | 0.01073 | 0.01166 | 0.00969 | 0.01756 | 0.01297 | 0.00409 | 0.00783 | 0.01622 | 0.00816 | 0.01073 | 0.00475 | 9.E-01 | 7.E-01 | 6.E-01 |

**Table S4A.** Relative abundance of *N*-glycans from CRL cell line.

| N-glycan | Theoretical m/z | Observed m/z | Mass Accuracy (ppm) | DMEM_1 | DMEM_2 | DMEM_3 | Avg | StdV | EMEM_1 | EMEM_2 | EMEM_3 | Avg | StdV | Plasmax_1 | Plasmax_2 | Plasmax_3 | Avg | StdV | P value DMEM EMEM | P value DMEM Plasmax | P value EMEM Plasmax |
|---|---|---|---|---|---|---|---|---|---|---|---|---|---|---|---|---|---|---|---|---|---|
| 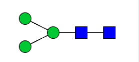 | 1165.6331 | 1165.6291 | 3.43 | 0.21055 | 0.18422 | 0.21313 | 0.20263 | 0.01599 | 0.17248 | 0.19654 | 0.16777 | 0.17893 | 0.01543 | 0.16628 | 0.18673 | 0.20616 | 0.18639 | 0.01994 | 1.E-01 | 3.E-01 | 6.E-01 |
| 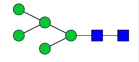 | 787.4203 | 787.4170 | 4.19 | 0.09155 | 0.08322 | 0.06792 | 0.08090 | 0.01199 | 0.07327 | 0.07669 | 0.08165 | 0.07720 | 0.00421 | 0.09355 | 0.08259 | 0.07849 | 0.08488 | 0.00778 | 6.E-01 | 7.E-01 | 2.E-01 |
| 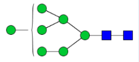 | 991.5201 | 991.5171 | 3.03 | 0.04261 | 0.03763 | 0.04168 | 0.04064 | 0.00265 | 0.04329 | 0.03566 | 0.03786 | 0.03894 | 0.00393 | 0.04423 | 0.04238 | 0.04119 | 0.04260 | 0.00153 | 6.E-01 | 3.E-01 | 2.E-01 |
| 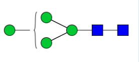 | 1369.7329 | 1369.7280 | 3.58 | 0.03597 | 0.04067 | 0.02885 | 0.03516 | 0.00595 | 0.01737 | 0.02186 | 0.02849 | 0.02257 | 0.00559 | 0.02268 | 0.02518 | 0.02763 | 0.02516 | 0.00247 | 6.E-02 | 5.E-02 | 5.E-01 |
| 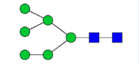 | 889.4702 | 889.4676 | 2.92 | 0.10993 | 0.08923 | 0.08996 | 0.09637 | 0.01175 | 0.10608 | 0.10047 | 0.11217 | 0.10624 | 0.00585 | 0.12566 | 0.12052 | 0.10540 | 0.11719 | 0.01053 | 3.E-01 | 8.E-02 | 2.E-01 |
| 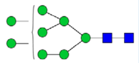 | 1093.5700 | 1093.5673 | 2.47 | 0.03858 | 0.03745 | 0.03831 | 0.03811 | 0.00059 | 0.05542 | 0.04198 | 0.04021 | 0.04587 | 0.00831 | 0.05115 | 0.05328 | 0.04768 | 0.05070 | 0.00283 | 2.E-01 | 2.E-03 | 4.E-01 |
| 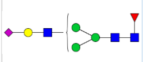 | 1075.5650 | 1075.5610 | 3.72 | 0.01138 | 0.01282 | 0.01036 | 0.01152 | 0.00123 | 0.01562 | 0.01489 | 0.01394 | 0.01482 | 0.00084 | 0.00967 | 0.01035 | 0.00801 | 0.00934 | 0.00120 | 2.E-02 | 9.E-02 | 3.E-03 |
| 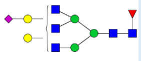 | 948.8301 | 948.8270 | 3.27 | 0.00594 | 0.00641 | 0.00699 | 0.00645 | 0.00053 | 0.00694 | 0.00633 | 0.00504 | 0.00610 | 0.00097 | 0.00794 | 0.00932 | 0.00879 | 0.00869 | 0.00070 | 6.E-01 | 1.E-02 | 2.E-02 |
| 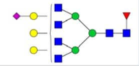 | 1098.5721 | 1098.5686 | 3.22 | 0.00343 | 0.00467 | 0.00455 | 0.00422 | 0.00069 | 0.00215 | 0.00312 | 0.00254 | 0.00261 | 0.00049 | 0.00469 | 0.00585 | 0.00583 | 0.00546 | 0.00067 | 3.E-02 | 9.E-02 | 4.E-03 |
| 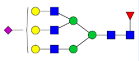 | 1016.8634 | 1016.8600 | 3.31 | 0.00090 | 0.00123 | 0.00113 | 0.00109 | 0.00017 | 0.00249 | 0.00255 | 0.00226 | 0.00243 | 0.00015 | 0.00130 | 0.00170 | 0.00147 | 0.00149 | 0.00020 | 5.E-04 | 5.E-02 | 3.E-03 |
| 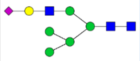 | 1192.6202 | 1192.6160 | 3.52 | 0.00082 | 0.00117 | 0.00112 | 0.00104 | 0.00019 | 0.00083 | 0.00093 | 0.00105 | 0.00094 | 0.00011 | 0.00000 | 0.00086 | 0.00070 | 0.00052 | 0.00046 | 5.E-01 | 1.E-01 | 2.E-01 |
| 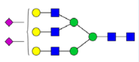 | 1079.2249 | 1079.2213 | 3.30 | 0.00036 | 0.00059 | 0.00052 | 0.00049 | 0.00012 | 0.00117 | 0.00121 | 0.00108 | 0.00116 | 0.00007 | 0.00166 | 0.00257 | 0.00232 | 0.00218 | 0.00047 | 1.E-03 | 4.E-03 | 2.E-02 |
| 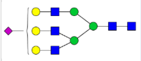 | 958.8336 | 958.8307 | 3.06 | 0.00000 | 0.00000 | 0.00000 | 0.00000 | 0.00000 | 0.00111 | 0.00119 | 0.00089 | 0.00107 | 0.00015 | 0.00112 | 0.00154 | 0.00126 | 0.00131 | 0.00022 | 3.E-04 | 5.E-04 | 2.E-01 |
| 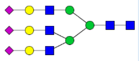 | 1199.6161 | 1199.6127 | 2.83 | 0.00000 | 0.00000 | 0.00000 | 0.00000 | 0.00000 | 0.00000 | 0.00000 | 0.00000 | 0.00000 | 0.00000 | 0.00060 | 0.00097 | 0.00078 | 0.00079 | 0.00018 | - | 2.E-03 | 2.E-03 |
| 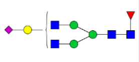 | 1198.1282 | 1198.1240 | 3.46 | 0.00000 | 0.00000 | 0.00000 | 0.00000 | 0.00000 | 0.00096 | 0.00099 | 0.00075 | 0.00090 | 0.00013 | 0.00000 | 0.00000 | 0.00000 | 0.00000 | 0.00000 | 3.E-04 | - | 3.E-04 |
| 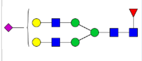 | 1300.1781 | 1300.1739 | 3.19 | 0.01183 | 0.01454 | 0.01454 | 0.01364 | 0.00157 | 0.03183 | 0.03033 | 0.03108 | 0.03108 | 0.00075 | 0.02673 | 0.02514 | 0.02546 | 0.02578 | 0.00084 | 6.E-05 | 3.E-04 | 1.E-03 |
| 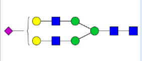 | 1213.1335 | 1213.1286 | 4.00 | 0.00816 | 0.00979 | 0.00910 | 0.00902 | 0.00082 | 0.02149 | 0.02030 | 0.01932 | 0.02037 | 0.00109 | 0.02082 | 0.02131 | 0.01690 | 0.01967 | 0.00242 | 1.E-04 | 2.E-03 | 7.E-01 |
| 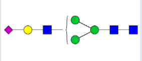 | 988.5204 | 988.5166 | 3.84 | 0.00498 | 0.00523 | 0.00427 | 0.00483 | 0.00050 | 0.00944 | 0.00939 | 0.00891 | 0.00925 | 0.00030 | 0.00615 | 0.00794 | 0.00656 | 0.00688 | 0.00093 | 2.E-04 | 3.E-02 | 1.E-02 |
| 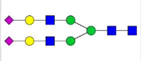 | 929.4828 | 929.4794 | 3.69 | 0.00188 | 0.00245 | 0.00300 | 0.00244 | 0.00056 | 0.00371 | 0.00344 | 0.00359 | 0.00358 | 0.00013 | 0.00474 | 0.00526 | 0.00446 | 0.00482 | 0.00040 | 3.E-02 | 4.E-03 | 7.E-03 |
| 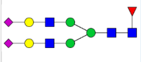 | 987.5126 | 987.5094 | 3.21 | 0.00148 | 0.00194 | 0.00149 | 0.00163 | 0.00026 | 0.00539 | 0.00514 | 0.00476 | 0.00510 | 0.00032 | 0.00500 | 0.00712 | 0.00566 | 0.00593 | 0.00108 | 1.E-04 | 3.E-03 | 3.E-01 |

| N-glycan | Theoretical m/z | Observed m/z | Mass Accuracy (ppm) | DMEM_1 | DMEM_2 | DMEM_3 | Avg | StdV | EMEM_1 | EMEM_2 | EMEM_3 | Avg | StdV | Plasmax_1 | Plasmax_2 | Plasmax_3 | Avg | StdV | P value DMEM EMEM | P value DMEM Plasmax | P value EMEM Plasmax |
|---|---|---|---|---|---|---|---|---|---|---|---|---|---|---|---|---|---|---|---|---|---|
| 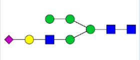 | 1090.5703 | 1090.5667 | 3.30 | 0.00125 | 0.00140 | 0.00098 | 0.00121 | 0.00021 | 0.00160 | 0.00192 | 0.00174 | 0.00175 | 0.00016 | 0.00173 | 0.00177 | 0.00180 | 0.00177 | 0.00003 | 3.E-02 | 1.E-02 | 9.E-01 |
| 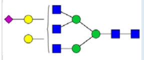 | 890.8004 | 890.7973 | 3.44 | 0.00106 | 0.00153 | 0.00155 | 0.00138 | 0.00028 | 0.00147 | 0.00136 | 0.00151 | 0.00145 | 0.00008 | 0.00170 | 0.00185 | 0.00195 | 0.00183 | 0.00013 | 7.E-01 | 6.E-02 | 1.E-02 |
| 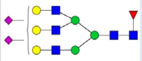 | 1137.2546 | 1137.2505 | 3.61 | 0.00047 | 0.00133 | 0.00113 | 0.00098 | 0.00045 | 0.00164 | 0.00172 | 0.00170 | 0.00169 | 0.00004 | 0.00245 | 0.00217 | 0.00269 | 0.00244 | 0.00026 | 5.E-02 | 8.E-03 | 8.E-03 |
| 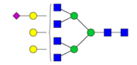 | 1040.5424 | 1040.5392 | 3.08 | 0.00000 | 0.00000 | 0.00000 | 0.00000 | 0.00000 | 0.00000 | 0.00000 | 0.00000 | 0.00000 | 0.00000 | 0.00000 | 0.00069 | 0.00063 | 0.00044 | 0.00038 | - | 1.E-01 | 1.E-01 |
| 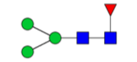 | 1339.7223 | 1339.7176 | 3.51 | 0.23384 | 0.24774 | 0.25452 | 0.24537 | 0.01054 | 0.12348 | 0.12649 | 0.14056 | 0.13018 | 0.00912 | 0.15465 | 0.15426 | 0.17760 | 0.16217 | 0.01336 | 1.E-04 | 1.E-03 | 3.E-02 |
| 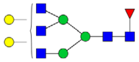 | 1242.1544 | 1242.1496 | 3.82 | 0.03353 | 0.03727 | 0.04211 | 0.03764 | 0.00430 | 0.02791 | 0.02208 | 0.02842 | 0.02614 | 0.00353 | 0.04433 | 0.03223 | 0.03641 | 0.03766 | 0.00614 | 2.E-02 | 1.E+00 | 5.E-02 |
| 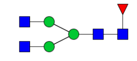 | 915.4914 | 915.4878 | 3.93 | 0.01821 | 0.02321 | 0.02299 | 0.02147 | 0.00282 | 0.05584 | 0.05662 | 0.05456 | 0.05567 | 0.00104 | 0.03683 | 0.03326 | 0.03029 | 0.03346 | 0.00327 | 4.E-05 | 9.E-03 | 4.E-04 |
| 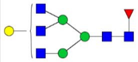 | 1140.1045 | 1140.0999 | 3.99 | 0.01689 | 0.01943 | 0.02178 | 0.01937 | 0.00245 | 0.00949 | 0.00878 | 0.00877 | 0.00902 | 0.00041 | 0.01862 | 0.01646 | 0.01548 | 0.01685 | 0.00161 | 2.E-03 | 2.E-01 | 1.E-03 |
| 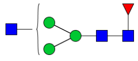 | 792.9283 | 792.9250 | 4.10 | 0.01652 | 0.02045 | 0.01932 | 0.01877 | 0.00203 | 0.04517 | 0.04538 | 0.04730 | 0.04595 | 0.00118 | 0.01903 | 0.01891 | 0.01770 | 0.01855 | 0.00073 | 4.E-05 | 9.E-01 | 4.E-06 |
| 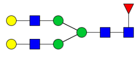 | 1119.5912 | 1119.5871 | 3.66 | 0.00778 | 0.00933 | 0.00920 | 0.00877 | 0.00086 | 0.01919 | 0.01747 | 0.01683 | 0.01783 | 0.00122 | 0.01041 | 0.00270 | 0.00381 | 0.00564 | 0.00417 | 5.E-04 | 3.E-01 | 8.E-03 |
| 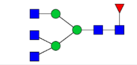 | 1038.0546 | 1038.0507 | 3.71 | 0.00752 | 0.00913 | 0.00875 | 0.00847 | 0.00084 | 0.01451 | 0.01403 | 0.01252 | 0.01369 | 0.00104 | 0.01081 | 0.00913 | 0.00827 | 0.00940 | 0.00129 | 2.E-03 | 4.E-01 | 1.E-02 |
| 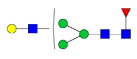 | 894.9782 | 894.9747 | 3.85 | 0.00649 | 0.00738 | 0.00527 | 0.00638 | 0.00106 | 0.00828 | 0.00853 | 0.00867 | 0.00849 | 0.00020 | 0.00584 | 0.00536 | 0.00499 | 0.00540 | 0.00043 | 3.E-02 | 2.E-01 | 3.E-04 |
| 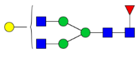 | 1017.5413 | 1017.5377 | 3.54 | 0.00278 | 0.00349 | 0.00298 | 0.00308 | 0.00037 | 0.01093 | 0.01047 | 0.00895 | 0.01012 | 0.00104 | 0.00322 | 0.00432 | 0.00332 | 0.00362 | 0.00061 | 4.E-04 | 3.E-01 | 7.E-04 |
| 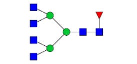 | 1160.6177 | 1160.6134 | 3.70 | 0.00235 | 0.00346 | 0.00305 | 0.00295 | 0.00056 | 0.00769 | 0.00799 | 0.00719 | 0.00762 | 0.00040 | 0.00451 | 0.00397 | 0.00367 | 0.00405 | 0.00042 | 3.E-04 | 5.E-02 | 4.E-04 |
| 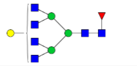 | 1262.6676 | 1262.6631 | 3.56 | 0.00091 | 0.00118 | 0.00122 | 0.00111 | 0.00017 | 0.00115 | 0.00137 | 0.00125 | 0.00126 | 0.00011 | 0.00103 | 0.00087 | 0.00069 | 0.00086 | 0.00017 | 3.E-01 | 2.E-01 | 3.E-02 |
| 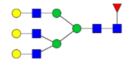 | 896.4721 | 896.4687 | 3.83 | 0.00046 | 0.00053 | 0.00063 | 0.00054 | 0.00008 | 0.00121 | 0.00112 | 0.00114 | 0.00115 | 0.00004 | 0.00000 | 0.00000 | 0.00000 | 0.00000 | 0.00000 | 3.E-04 | 4.E-04 | 1.E-06 |
| 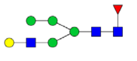 | 997.0281 | 997.0246 | 3.46 | 0.00069 | 0.00073 | 0.00048 | 0.00063 | 0.00013 | 0.00071 | 0.00075 | 0.00068 | 0.00071 | 0.00003 | 0.00000 | 0.00000 | 0.00000 | 0.00000 | 0.00000 | 4.E-01 | 1.E-03 | 3.E-06 |
| 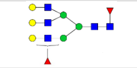 | 954.5019 | 954.5021 | 0.24 | 0.00065 | 0.00046 | 0.00065 | 0.00059 | 0.00011 | 0.00000 | 0.00000 | 0.00000 | 0.00000 | 0.00000 | 0.00000 | 0.00000 | 0.00000 | 0.00000 | 0.00000 | 7.E-04 | 7.E-04 | - |
| 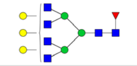 | 978.1809 | 978.1783 | 2.66 | 0.00218 | 0.00255 | 0.00318 | 0.00264 | 0.00050 | 0.00405 | 0.00365 | 0.00244 | 0.00338 | 0.00084 | 0.00433 | 0.00355 | 0.00342 | 0.00377 | 0.00049 | 3.E-01 | 5.E-02 | 5.E-01 |
| 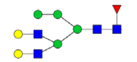 | 1221.6411 | 1221.6424 | 1.06 | 0.00122 | 0.00097 | 0.00126 | 0.00115 | 0.00016 | 0.00278 | 0.00201 | 0.00276 | 0.00252 | 0.00044 | 0.00309 | 0.00166 | 0.00122 | 0.00199 | 0.00097 | 7.E-03 | 2.E-01 | 4.E-01 |
| 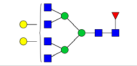 | 910.1476 | 910.1441 | 3.88 | 0.00064 | 0.00094 | 0.00054 | 0.00071 | 0.00021 | 0.00081 | 0.00089 | 0.00066 | 0.00079 | 0.00012 | 0.00071 | 0.00000 | 0.00075 | 0.00049 | 0.00042 | 6.E-01 | 5.E-01 | 3.E-01 |

| N-glycan | Theoretical m/z | Observed m/z | Mass Accuracy (ppm) | DMEM_1 | DMEM_2 | DMEM_3 | Avg | StdV | EMEM_1 | EMEM_2 | EMEM_3 | Avg | StdV | Plasmax_1 | Plasmax_2 | Plasmax_3 | Avg | StdV | P value DMEM EMEM | P value DMEM Plasmax | P value EMEM Plasmax |
|---|---|---|---|---|---|---|---|---|---|---|---|---|---|---|---|---|---|---|---|---|---|
| 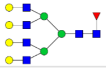 | 1046.2142 | 1046.2102 | 3.79 | 0.00052 | 0.00103 | 0.00104 | 0.00086 | 0.00030 | 0.00152 | 0.00160 | 0.00109 | 0.00141 | 0.00027 | 0.00114 | 0.00093 | 0.00104 | 0.00103 | 0.00010 | 8.E-02 | 4.E-01 | 9.E-02 |
| 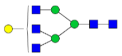 | 1053.0599 | 1053.0561 | 3.56 | 0.01212 | 0.01472 | 0.01273 | 0.01319 | 0.00136 | 0.00866 | 0.00760 | 0.00725 | 0.00784 | 0.00074 | 0.01627 | 0.01544 | 0.01318 | 0.01497 | 0.00160 | 4.E-03 | 2.E-01 | 2.E-03 |
| 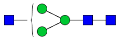 | 705.8837 | 705.8803 | 4.75 | 0.00789 | 0.00829 | 0.00701 | 0.00773 | 0.00066 | 0.01581 | 0.01917 | 0.01895 | 0.01798 | 0.00188 | 0.00727 | 0.00902 | 0.00750 | 0.00793 | 0.00095 | 9.E-04 | 8.E-01 | 1.E-03 |
| 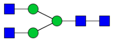 | 828.4468 | 828.4438 | 3.62 | 0.00781 | 0.00909 | 0.00817 | 0.00836 | 0.00066 | 0.02188 | 0.02339 | 0.02133 | 0.02220 | 0.00106 | 0.01141 | 0.01375 | 0.01268 | 0.01261 | 0.00117 | 4.E-05 | 5.E-03 | 5.E-04 |
| 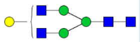 | 930.4967 | 930.4923 | 4.73 | 0.00223 | 0.00237 | 0.00215 | 0.00225 | 0.00011 | 0.00842 | 0.00880 | 0.00711 | 0.00811 | 0.00088 | 0.00249 | 0.00310 | 0.00259 | 0.00273 | 0.00033 | 3.E-04 | 7.E-02 | 6.E-04 |
| 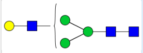 | 807.9336 | 807.9304 | 3.90 | 0.00770 | 0.00871 | 0.00602 | 0.00748 | 0.00136 | 0.00831 | 0.00859 | 0.00787 | 0.00826 | 0.00037 | 0.00533 | 0.00733 | 0.00596 | 0.00621 | 0.00102 | 4.E-01 | 3.E-01 | 3.E-02 |
| 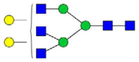 | 1155.1098 | 1155.1049 | 4.20 | 0.00386 | 0.00279 | 0.00132 | 0.00266 | 0.00128 | 0.00259 | 0.00348 | 0.00260 | 0.00289 | 0.00051 | 0.00488 | 0.00849 | 0.00434 | 0.00591 | 0.00226 | 8.E-01 | 1.E-01 | 9.E-02 |
| 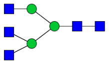 | 951.0100 | 951.0065 | 3.63 | 0.00285 | 0.00424 | 0.00153 | 0.00287 | 0.00136 | 0.00575 | 0.00585 | 0.00504 | 0.00555 | 0.00044 | 0.00384 | 0.00424 | 0.00372 | 0.00393 | 0.00027 | 3.E-02 | 3.E-01 | 6.E-03 |
| 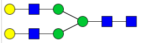 | 1032.5466 | 1032.5426 | 3.87 | 0.00220 | 0.00258 | 0.00223 | 0.00234 | 0.00021 | 0.00161 | 0.00000 | 0.00357 | 0.00173 | 0.00179 | 0.00428 | 0.00406 | 0.00460 | 0.00431 | 0.00027 | 6.E-01 | 6.E-04 | 7.E-02 |
| 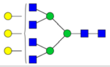 | 920.1512 | 920.1477 | 3.77 | 0.00103 | 0.00127 | 0.00129 | 0.00120 | 0.00014 | 0.00146 | 0.00151 | 0.00122 | 0.00140 | 0.00015 | 0.00218 | 0.00249 | 0.00219 | 0.00229 | 0.00017 | 2.E-01 | 1.E-03 | 3.E-03 |

**Table S4B.** Relative abundance of *O*-glycans from CRL cell line.

| HexNac-Hex-deHex-NeuAc | Theoretical m/z | Observed m/z | Mass Accuracy (ppm) | DMEM_1 | DMEM_2 | DMEM_3 | Avg | StdV | EMEM_1 | EMEM_2 | EMEM_3 | Avg | StdV | Plasmax_1 | Plasmax_2 | Plasmax_3 | Avg | StdV | P value DMEM EMEM | P value DMEM Plasmax | P value EMEM Plasmax |
|---|---|---|---|---|---|---|---|---|---|---|---|---|---|---|---|---|---|---|---|---|---|
| **1-1-0-1** | 857.4496 | 857.4486 | 1.17 | 0.00000 | 0.00000 | 0.00000 | 0.00000 | 0.00000 | 0.00000 | 0.00000 | 0.00000 | 0.00000 | 0.00000 | 0.11934 | 0.07092 | 0.08655 | 0.09227 | 0.02471 | - | 3.E-03 | 3.E-03 |
| **2-0-1-1** | 1072.5653 | 1072.5571 | 7.65 | 0.10652 | 0.04425 | 0.11048 | 0.08708 | 0.03715 | 0.37118 | 0.33991 | 0.32090 | 0.34400 | 0.02539 | 0.09332 | 0.02185 | 0.03067 | 0.04862 | 0.03897 | 6.E-04 | 3.E-01 | 4.E-04 |
| **2-2-0-0** | 945.5020 | 945.5003 | 1.80 | 0.00000 | 0.00000 | 0.00000 | 0.00000 | 0.00000 | 0.00000 | 0.00000 | 0.00000 | 0.00000 | 0.00000 | 0.07376 | 0.03149 | 0.02733 | 0.04419 | 0.02569 | - | 4.E-02 | 4.E-02 |
| **2-2-0-2** | 834.4287 | 834.4302 | 1.86 | 0.11649 | 0.11144 | 0.09427 | 0.10740 | 0.01164 | 0.07308 | 0.09308 | 0.16299 | 0.10972 | 0.04721 | 0.14829 | 0.09584 | 0.11341 | 0.11918 | 0.02670 | 9.E-01 | 5.E-01 | 8.E-01 |
| **2-3-0-1** | 755.8917 | 755.8985 | 9.00 | 0.00000 | 0.00000 | 0.00000 | 0.00000 | 0.00000 | 0.13479 | 0.09992 | 0.14667 | 0.12713 | 0.02430 | 0.00000 | 0.00000 | 0.00000 | 0.00000 | 0.00000 | 8.E-04 | - | 8.E-04 |
| **2-4-0-3** | 1219.1153 | 1219.1103 | 4.10 | 0.01152 | 0.00925 | 0.00956 | 0.01011 | 0.00123 | 0.00000 | 0.00000 | 0.00000 | 0.00000 | 0.00000 | 0.01196 | 0.01667 | 0.01286 | 0.01383 | 0.00250 | 1.E-04 | 8.E-02 | 7.E-04 |
| **2-4-3-2** | 1299.6623 | 1299.6696 | 5.66 | 0.00209 | 0.00409 | 0.00190 | 0.00269 | 0.00121 | 0.00000 | 0.00000 | 0.00000 | 0.00000 | 0.00000 | 0.00000 | 0.00000 | 0.00000 | 0.00000 | 0.00000 | 2.E-02 | 2.E-02 | - |
| **2-5-0-3** | 1321.1652 | 1321.1607 | 3.41 | 0.01505 | 0.01350 | 0.01747 | 0.01534 | 0.00200 | 0.00000 | 0.00000 | 0.00000 | 0.00000 | 0.00000 | 0.00704 | 0.01043 | 0.00965 | 0.00904 | 0.00178 | 2.E-04 | 2.E-02 | 9.E-04 |
| **2-5-1-2** | 1227.6230 | 1227.6227 | 0.20 | 0.01076 | 0.01717 | 0.00629 | 0.01141 | 0.00547 | 0.00000 | 0.00000 | 0.00000 | 0.00000 | 0.00000 | 0.01134 | 0.01526 | 0.01138 | 0.01266 | 0.00225 | 2.E-02 | 7.E-01 | 6.E-04 |
| **2-5-3-0** | 1040.5385 | 1040.5296 | 8.51 | 0.01718 | 0.01381 | 0.01593 | 0.01564 | 0.00170 | 0.00839 | 0.00880 | 0.01233 | 0.00984 | 0.00217 | 0.00957 | 0.00900 | 0.00475 | 0.00777 | 0.00264 | 2.E-02 | 1.E-02 | 4.E-01 |
| **2-6-0-0** | 881.4546 | 881.4550 | 0.51 | 0.04208 | 0.05225 | 0.03547 | 0.04327 | 0.00846 | 0.00000 | 0.00000 | 0.00000 | 0.00000 | 0.00000 | 0.05210 | 0.06255 | 0.02096 | 0.04520 | 0.02163 | 9.E-04 | 9.E-01 | 2.E-02 |
| **2-7-0-0** | 983.5045 | 983.5003 | 4.22 | 0.07074 | 0.09787 | 0.06330 | 0.07730 | 0.01820 | 0.00000 | 0.00000 | 0.00000 | 0.00000 | 0.00000 | 0.05895 | 0.09481 | 0.06823 | 0.07400 | 0.01861 | 2.E-03 | 8.E-01 | 2.E-03 |
| **2-8-0-0** | 1085.5544 | 1085.5504 | 3.64 | 0.14158 | 0.19399 | 0.15193 | 0.16250 | 0.02776 | 0.09054 | 0.10621 | 0.06935 | 0.08870 | 0.01850 | 0.11576 | 0.19044 | 0.17486 | 0.16035 | 0.03939 | 2.E-02 | 9.E-01 | 5.E-02 |
| **2-9-0-0** | 1187.6043 | 1187.5997 | 3.83 | 0.09194 | 0.11829 | 0.11604 | 0.10876 | 0.01461 | 0.06618 | 0.08794 | 0.04565 | 0.06659 | 0.02115 | 0.05644 | 0.09409 | 0.10082 | 0.08378 | 0.02391 | 5.E-02 | 2.E-01 | 4.E-01 |
| **2-10-0-0** | 1289.6542 | 1289.6502 | 3.06 | 0.02075 | 0.02228 | 0.02539 | 0.02281 | 0.00236 | 0.00000 | 0.00000 | 0.00000 | 0.00000 | 0.00000 | 0.01053 | 0.01993 | 0.01168 | 0.01405 | 0.00513 | 8.E-05 | 5.E-02 | 9.E-03 |
| **3-1-2-2** | 1029.0311 | 1029.0233 | 7.58 | 0.01224 | 0.00866 | 0.00962 | 0.01018 | 0.00185 | 0.00000 | 0.00000 | 0.00000 | 0.00000 | 0.00000 | 0.00848 | 0.00358 | 0.00000 | 0.00402 | 0.00425 | 7.E-04 | 8.E-02 | 2.E-01 |
| **3-3-1-1** | 965.4995 | 965.4937 | 5.96 | 0.04284 | 0.05107 | 0.03954 | 0.04448 | 0.00594 | 0.00000 | 0.00000 | 0.00000 | 0.00000 | 0.00000 | 0.00880 | 0.02624 | 0.01890 | 0.01798 | 0.00875 | 2.E-04 | 1.E-02 | 2.E-02 |
| **3-3-1-3** | 1326.6732 | 1326.6718 | 1.02 | 0.01909 | 0.01139 | 0.01911 | 0.01653 | 0.00445 | 0.01878 | 0.01678 | 0.01341 | 0.01632 | 0.00271 | 0.00000 | 0.00000 | 0.00000 | 0.00000 | 0.00000 | 9.E-01 | 3.E-03 | 5.E-04 |
| **3-3-2-2** | 1233.1309 | 1233.1227 | 6.65 | 0.03943 | 0.02948 | 0.03357 | 0.03416 | 0.00500 | 0.03478 | 0.03209 | 0.02517 | 0.03068 | 0.00496 | 0.01972 | 0.02283 | 0.02229 | 0.02161 | 0.00166 | 4.E-01 | 1.E-02 | 4.E-02 |
| **3-4-1-1** | 1067.5494 | 1067.5441 | 4.92 | 0.03319 | 0.01316 | 0.02023 | 0.02219 | 0.01016 | 0.01487 | 0.03276 | 0.02275 | 0.02346 | 0.00897 | 0.01281 | 0.03787 | 0.03338 | 0.02802 | 0.01336 | 9.E-01 | 6.E-01 | 6.E-01 |
| **3-5-1-1** | 1169.5993 | 1169.5921 | 6.11 | 0.01417 | 0.01931 | 0.01861 | 0.01736 | 0.00279 | 0.00767 | 0.00910 | 0.01123 | 0.00933 | 0.00179 | 0.00566 | 0.01422 | 0.01581 | 0.01190 | 0.00546 | 1.E-02 | 2.E-01 | 5.E-01 |
| **4-4-0-3** | 976.4970 | 976.4917 | 5.46 | 0.00654 | 0.00627 | 0.00899 | 0.00727 | 0.00150 | 0.01246 | 0.00780 | 0.00704 | 0.00910 | 0.00294 | 0.00225 | 0.00412 | 0.00994 | 0.00544 | 0.00401 | 4.E-01 | 5.E-01 | 3.E-01 |
| **4-9-0-0** | 955.4897 | 955.4866 | 3.21 | 0.00445 | 0.00503 | 0.00846 | 0.00598 | 0.00217 | 0.00000 | 0.00000 | 0.00000 | 0.00000 | 0.00000 | 0.00330 | 0.00439 | 0.01799 | 0.00856 | 0.00818 | 9.E-03 | 6.E-01 | 1.E-01 |
| **5-5-0-1** | 1327.6810 | 1327.6714 | 7.19 | 0.00306 | 0.00551 | 0.00223 | 0.00360 | 0.00171 | 0.00308 | 0.00587 | 0.00395 | 0.00430 | 0.00143 | 0.00199 | 0.00233 | 0.00320 | 0.00250 | 0.00062 | 6.E-01 | 4.E-01 | 1.E-01 |
| **5-5-1-0** | 1234.1387 | 1234.1275 | 9.08 | 0.00489 | 0.00518 | 0.00620 | 0.00542 | 0.00069 | 0.00000 | 0.00000 | 0.00000 | 0.00000 | 0.00000 | 0.00548 | 0.00434 | 0.00407 | 0.00463 | 0.00075 | 2.E-04 | 2.E-01 | 4.E-04 |
| **6-3-1-0** | 1152.6021 | 1152.5974 | 4.03 | 0.02459 | 0.02037 | 0.02539 | 0.02345 | 0.00269 | 0.02755 | 0.03101 | 0.02480 | 0.02779 | 0.00311 | 0.01279 | 0.01514 | 0.00948 | 0.01247 | 0.00284 | 1.E-01 | 8.E-03 | 3.E-03 |
| **6-4-0-1** | 1348.1942 | 1348.1842 | 7.42 | 0.04674 | 0.04763 | 0.05414 | 0.04950 | 0.00404 | 0.03001 | 0.02480 | 0.02849 | 0.02777 | 0.00268 | 0.01008 | 0.00763 | 0.00951 | 0.00907 | 0.00128 | 1.E-03 | 8.E-05 | 4.E-04 |
| **6-4-1-0** | 1254.6520 | 1254.6478 | 3.31 | 0.05771 | 0.04319 | 0.05420 | 0.05170 | 0.00758 | 0.05257 | 0.05474 | 0.04422 | 0.05051 | 0.00555 | 0.02869 | 0.03563 | 0.03797 | 0.03410 | 0.00483 | 8.E-01 | 3.E-02 | 2.E-02 |
| **6-5-0-1** | 967.1654 | 967.1707 | 5.51 | 0.01670 | 0.01543 | 0.01914 | 0.01709 | 0.00189 | 0.02057 | 0.02015 | 0.01295 | 0.01789 | 0.00428 | 0.10971 | 0.08623 | 0.13941 | 0.11178 | 0.02665 | 8.E-01 | 4.E-03 | 4.E-03 |
| **6-5-1-0** | 904.8039 | 904.8009 | 3.28 | 0.02640 | 0.01808 | 0.03041 | 0.02496 | 0.00629 | 0.03049 | 0.02557 | 0.04500 | 0.03369 | 0.01010 | 0.00000 | 0.00000 | 0.00000 | 0.00000 | 0.00000 | 3.E-01 | 2.E-03 | 4.E-03 |
| **6-6-0-1** | 1035.1986 | 1035.1938 | 4.67 | 0.00127 | 0.00205 | 0.00212 | 0.00181 | 0.00047 | 0.00298 | 0.00348 | 0.00311 | 0.00319 | 0.00026 | 0.00185 | 0.00218 | 0.00491 | 0.00298 | 0.00168 | 1.E-02 | 3.E-01 | 8.E-01 |

Figure S1

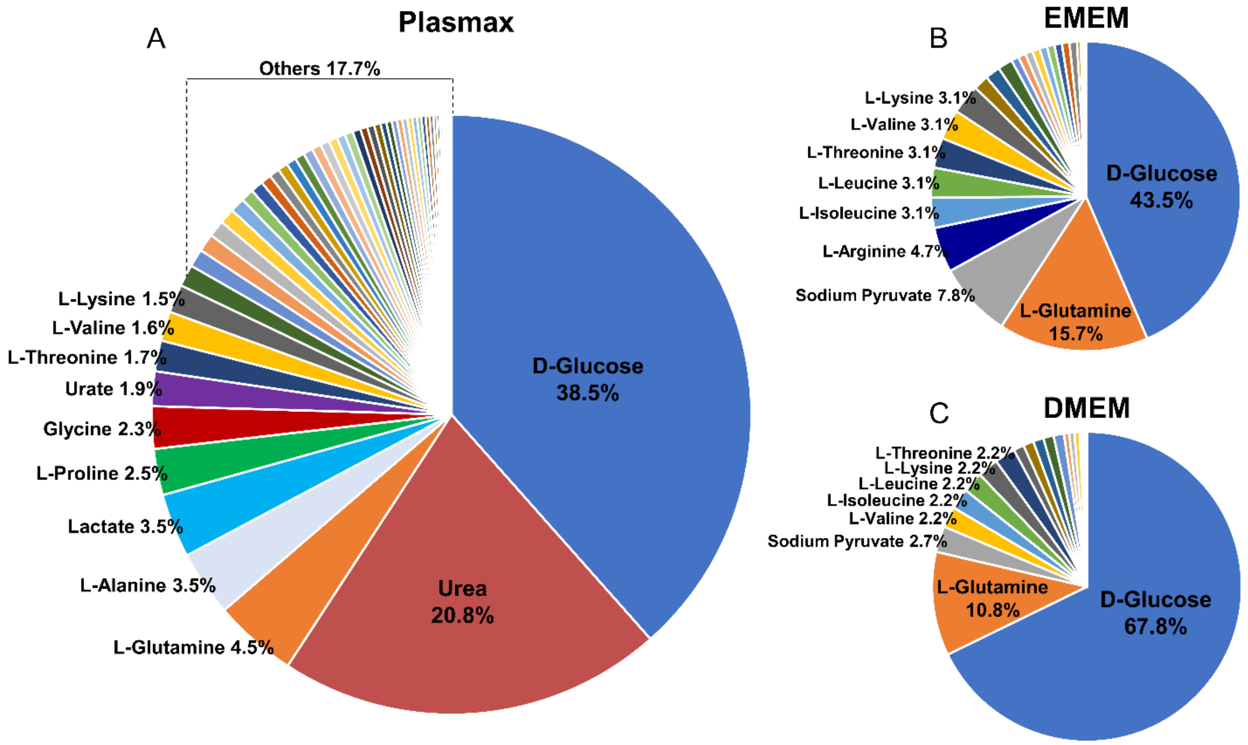


**Figure S1.** Nutritional composition of three media: **(A)** Plasmax, **(B)** EMEM, and **(C)** DMEM.

**Figure S2**

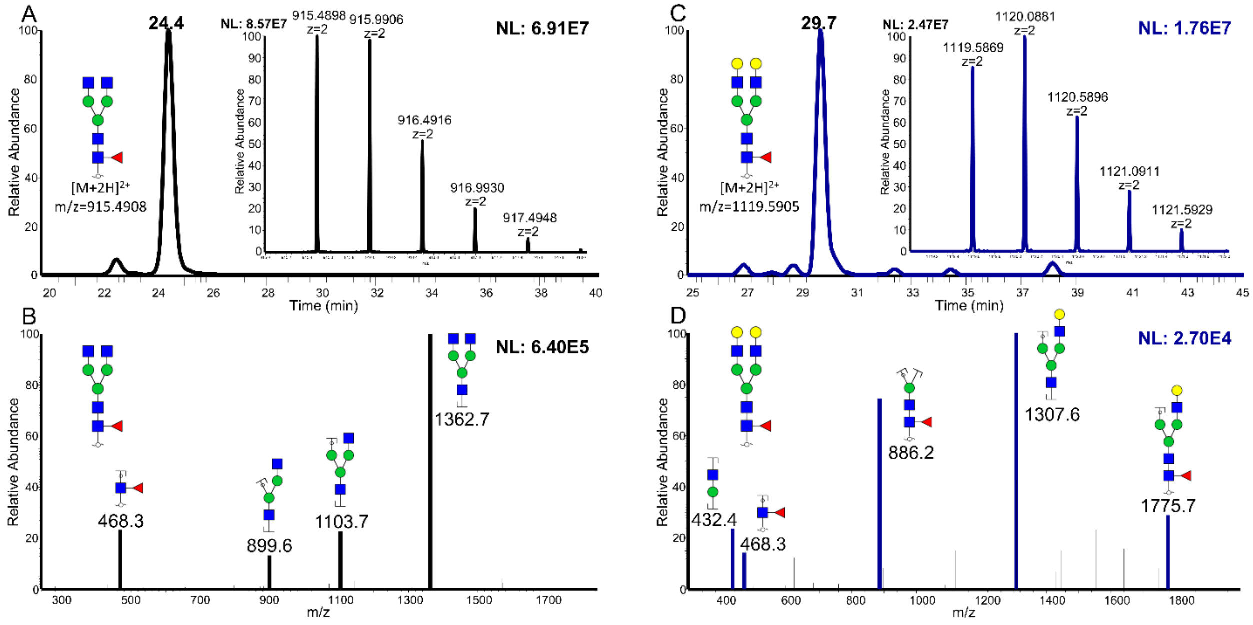


**Figure S2.** Examples of positional structural identification method of **(A)** *N*-glycan HexNAc$_4$Hex$_3$DeoxyHex$_1$ from 231BR cell line and **(C)** *N*-glycan HexNAc4Hex5DeoxyHex1 from CRL cell line using LC-MS/MS. Insets of **(A)** and **(C)** are the full MS spectra, **(B)** and **(D)** are MS/MS spectra with identified fragment ions assigned to corresponding peaks. Symbols: ■, N-acetylglucosamine (GlcNAc); ○, Galactose (Gal); ▼, Fucose (Fuc); ●, Mannose (Man).

**Figure S3**

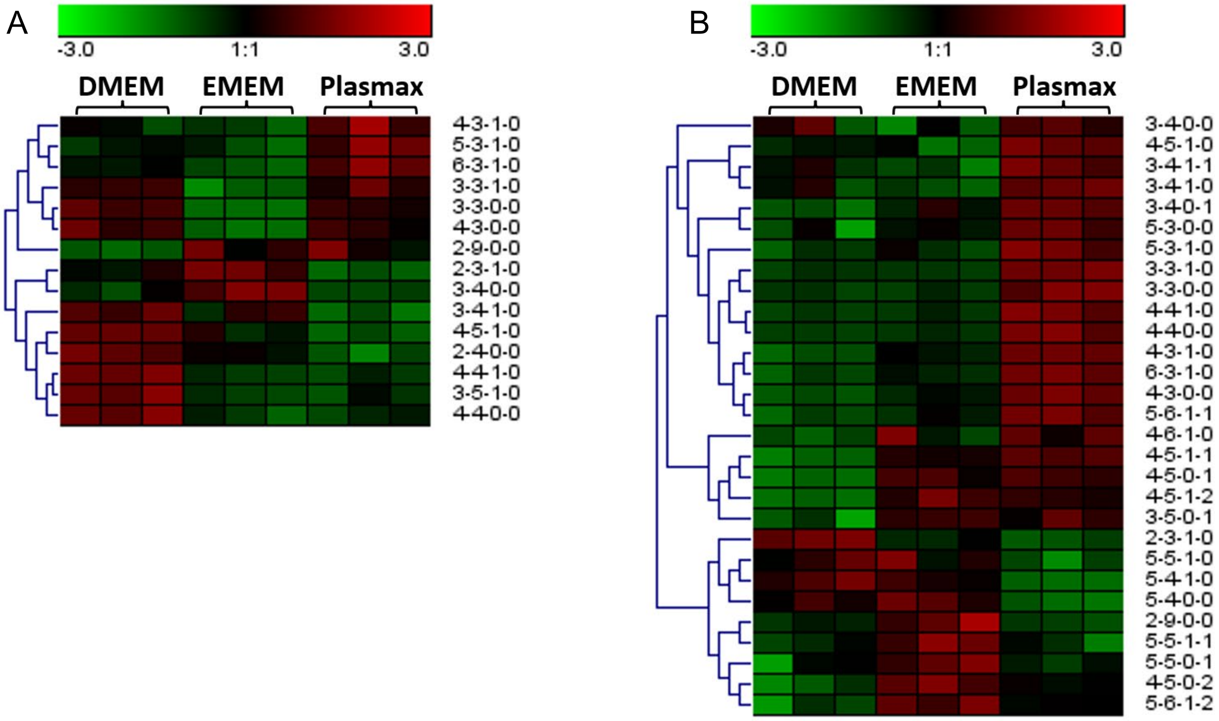


**Figure S3.** Heatmaps of significant *N*-glycans from **(A)** 231BR cell line and **(B)** CRL cell line. Red color denotes up-regulation, green color denotes down-regulation.

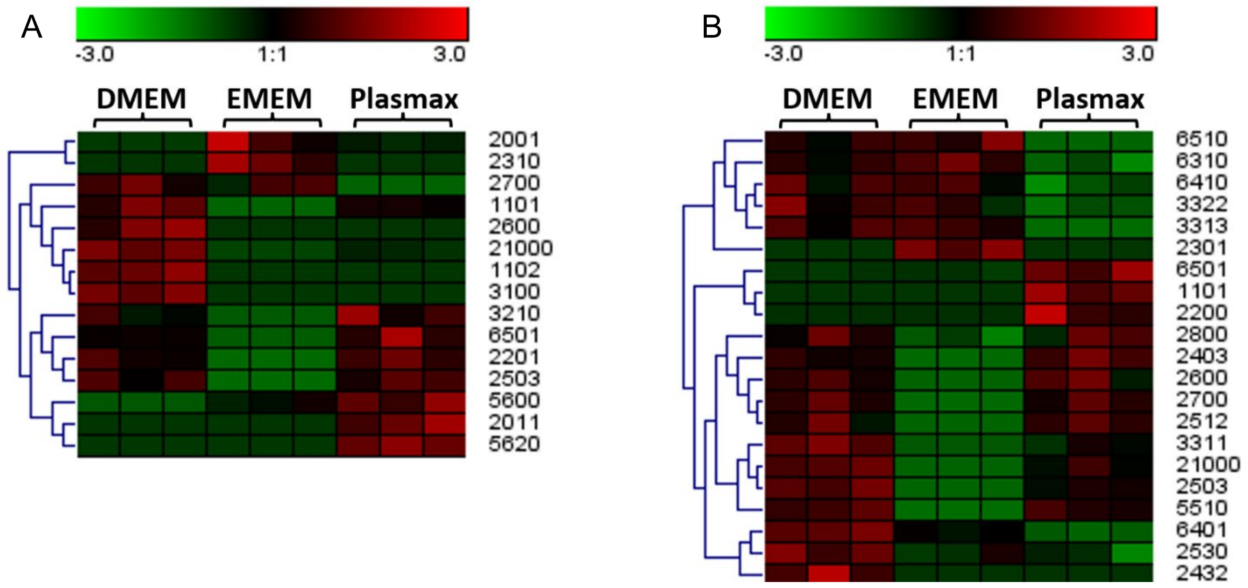


**Figure S4.** Heatmaps of significant *O*-glycans from **(A)** 231BR cell line and **(B)** CRL cell line. Red color denotes up-regulation, green color denotes down-regulation.

# Figure S5

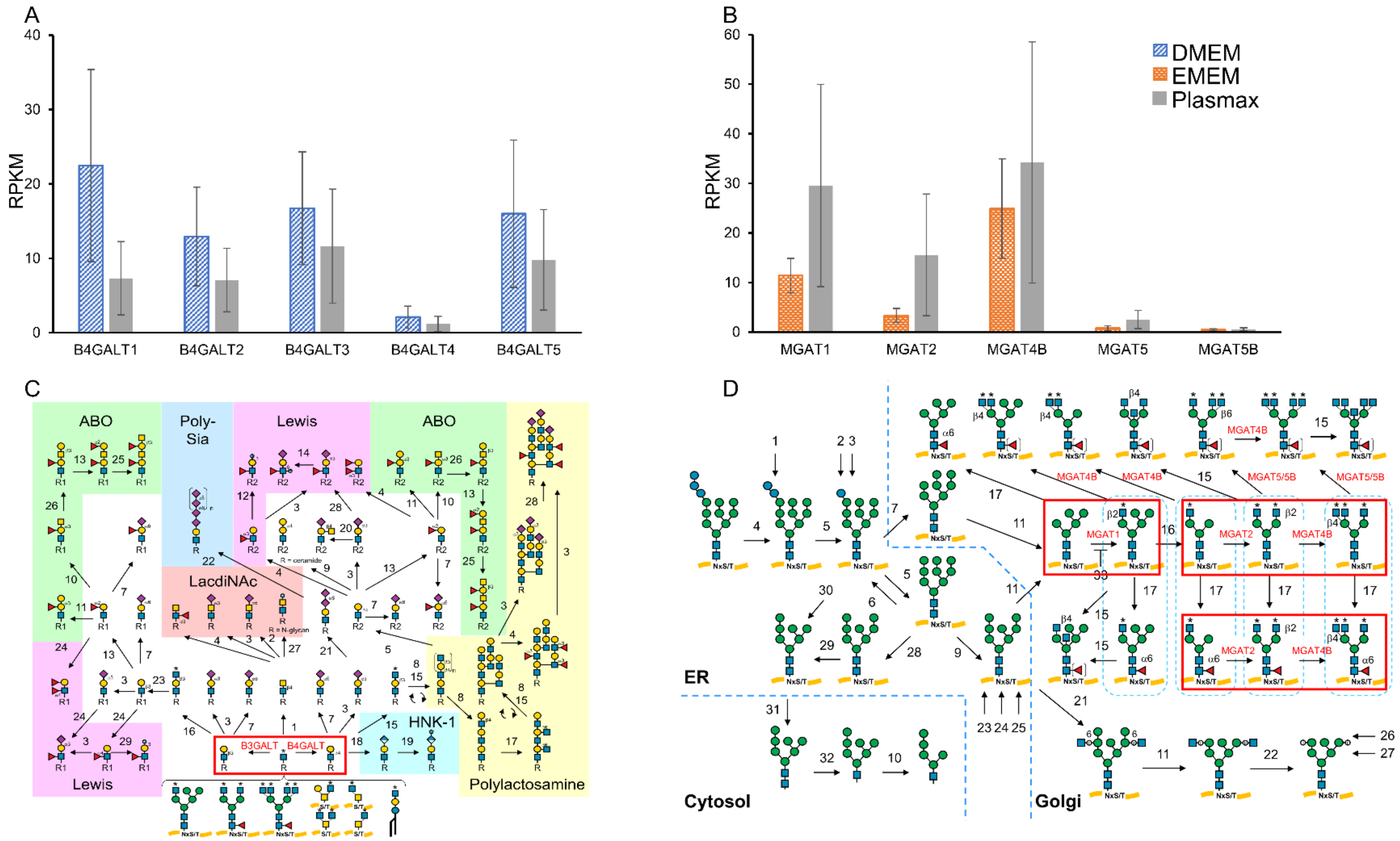


**Figure S5.** Transcript expressions of 231BR cell line. **(A)** Galactosyltransferases in DMEM and Plasmax; **(B)** *N*-acetylglucosaminyltransferases in EMEM and Plasmax. **(C)** Synthesis pathways of complex capping *N*- and *O*-glycans. The red box highlights the processes of connecting galactose to GlcNAc, catalyzed by B4GALT/B3GALT. **(D)** Synthesis pathways of *N*-glycan processing and branching. Red boxes highlight the processes of attaching GlcNAc to mannose on core structure, catalyzed by MGATs. The synthesis pathways are acquired from GlycoMaple (https://glycosmos.org/glycomaple/index).

**Figure S6**

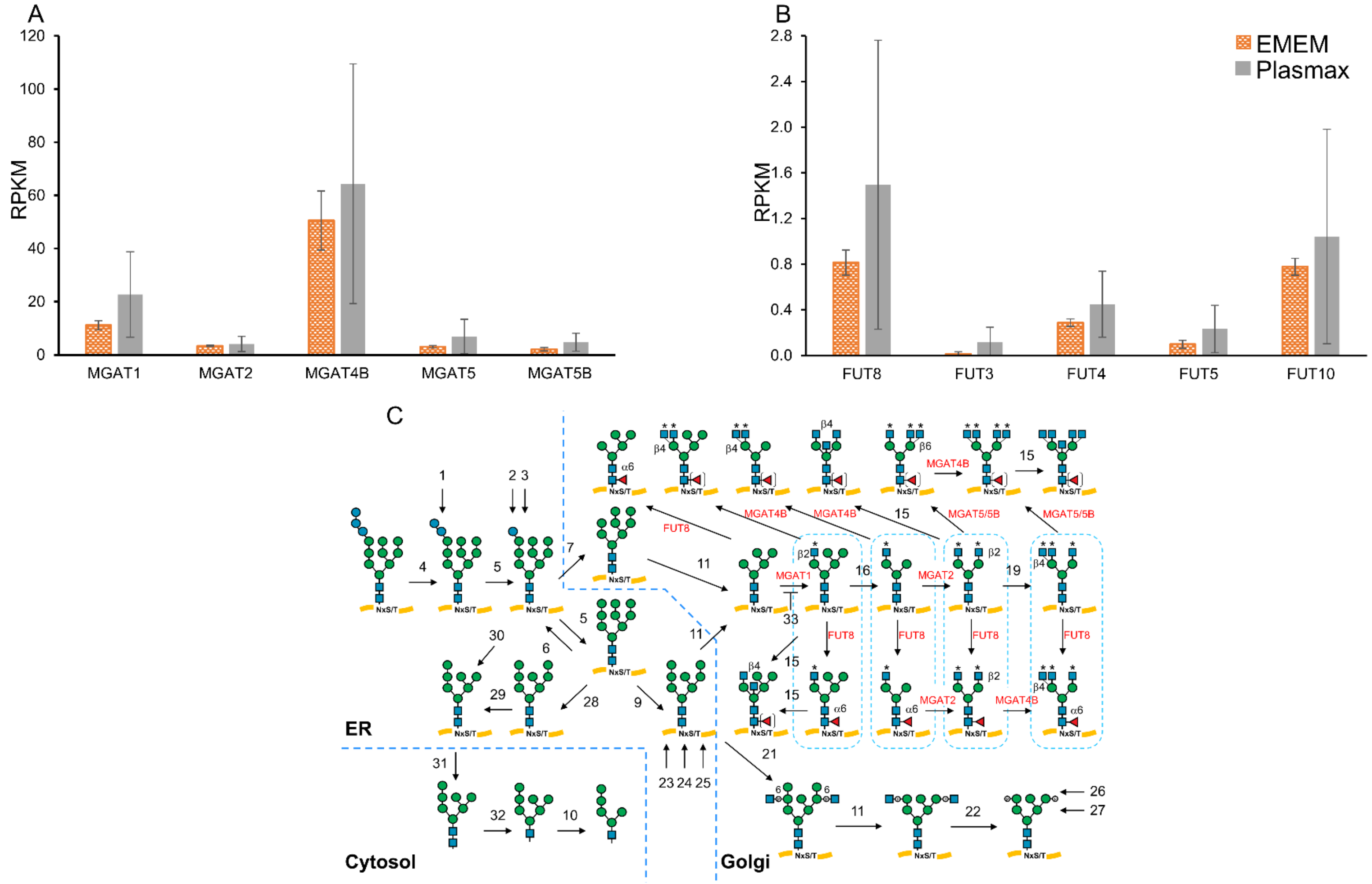


**Figure S6.** Transcript expressions of CRL cell line. **(A)** *N*-acetylglucosaminyltransferases in EMEM and Plasmax; **(B)** Fucosyltransferases in EMEM and Plasmax. **(C)** Synthesis pathways of *N*-glycan processing and branching. The labeled transcripts of transferases depict the processes of attaching GlcNAc to mannose on core structure, and core-fucosylation, catalyzed by MGATs, and FUT8, respectively. The synthesis pathways are acquired from GlycoMaple (https://glycosmos.org/glycomaple/index).

**Figure S7**

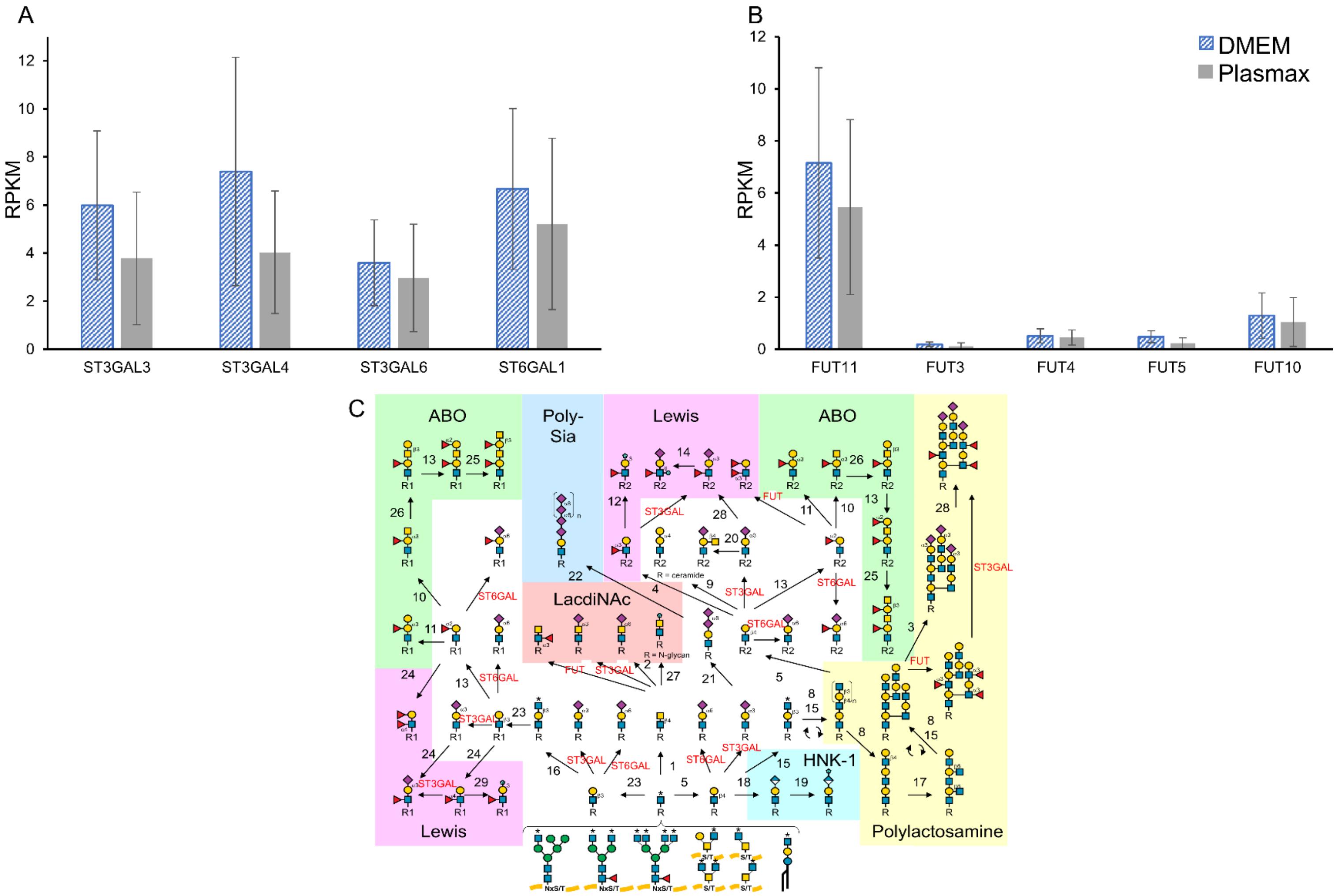


**Figure S7.** Transcript expressions of **(A)** Sialytransferases in 231BR cell line cultivated by DMEM and Plasmax; **(B)** Fucosyltransferases in CRL cell line cultivated by DMEM and Plasmax. **(C)** Synthesis pathways of complex capping *N*- and *O*-glycans. The labeled transcripts of transferases depict the processes of sialylation catalyzed by ST3GAL or ST6GAL, and fucosylation catalyzed by FUTs. The synthesis pathways are acquired from GlycoMaple (https://glycosmos.org/glycomaple/index).
